# Enhancer-based gene therapy: a new path for precision medicine

**DOI:** 10.1186/s41065-026-00655-0

**Published:** 2026-03-21

**Authors:** Hany E. Marei

**Affiliations:** Department of Cytology and Histology, Faculty of Veterinary Medicine, Mansoura University, Mansoura, 35116 Egypt

**Keywords:** Enhancer-based gene therapy, Cis-regulatory elements, Transcriptional regulation, Tissue- and cell-specific expression, Gene dosage control, Epigenetics, Synthetic biology, CRISPR-Cas9, Adeno-associated viruses (AAVs), Transgene expression, Neurodegenerative diseases, Cancer therapy, Functional genomics, Enhancer screening, Chromatin structure, Histone modifications, DNA looping, Transcriptional memory, Non-coding DNA, Multi-omics approaches, RNA-based therapies, Genome editing, Therapeutic precision, Spatiotemporal gene regulation

## Abstract

Enhancers are critical cis-regulatory elements that regulate gene expression in a context-dependent manner by integrating transcription factor binding, chromatin state, and the three-dimensional organization of the genome. Recent advances in functional genomics and synthetic biology have increased interest in harnessing enhancer activity to regulate the expression of therapeutic genes. Unlike traditional approaches that rely on promoter-driven gene regulation, enhancer-based approaches can bias transgene expression toward specific cellular states or disease contexts; however, this control remains probabilistic and highly dependent on the chromatin environment. This review summarizes current knowledge of enhancer biology, discusses new strategies for utilizing enhancer function directly, and examines the potential benefits and drawbacks of using enhancer-based strategies for gene therapy applications. Often delivered using adeno-associated virus (AAV) vectors with tailored capsids, enhancers in gene therapy can be included into expression cassettes. Astrocyte- or microglia-specific enhancers in the brain enable enriched or preferential distribution of neuroprotective or immunomodulatory genes, hence lowering unintentional expression in non-target cell types. It is important to control gene expression for specific cell types for the treatment of neurodegenerative conditions such as Alzheimer’s or Parkinson’s disease, were unintentional gene expression results in negative consequences. However, the uses of enhancer-guided gene therapy go beyond the central nervous system. In cancer, therapeutic constructs are designed to inhibit oncogenic expression or induce tumor suppression pathways, using enhancers in either malignant or immune cells as a target. Similarly, through the use of tissue-specific enhancers in cardiovascular and regenerative medicine, lineage-enriched genes can be used to promote repair of damaged tissues and enhance functional recovery. Enhancer-based systems that modulate the levels of gene expression (enhancer systems that adjust gene expression to levels that are physiologically appropriate for a given cell type) may also be useful in diseases caused by imbalances of gene dosage (e.g., haploinsufficiency and copy number variations). However, despite the potential promise of enhancer-driven gene therapy, many technical and translational hurdles remain. Mapping and validating the function of cell-type-specific enhancers is hampered by the dynamic, context-dependent regulation of chromatin. The identification of enhancers across a variety of developmental stages and clinical states is being accelerated through the combination of recent advances in single-cell epigenomic techniques (e.g., ATAC-seq, ChIP-seq, and multi-omic integration). Recent advances in non-viral delivery methods and AAV capsid engineering are improving the safety, efficacy, and scalability of enhancer-driven gene therapies. However, there must be careful regulatory oversight to avoid unintentional activation of enhancers and ensure continuing efficacy of enhancer-guided therapies. This paper provides an overview of the conceptual basis of enhancer-driven gene therapies, the currently available applications, and barriers to their clinical application. We show how the combination of delivery technology, synthetic biology, and genomics is enabling new possibilities for tailored gene therapy particular to cell- and disease-specific. Enhancer-driven gene therapy could become an important component of next-generation precision medicine by addressing current challenges and using creative technology.

## Introduction

Gene therapy is a hopeful new type of medical treatment that can be utilized to replace, modify and/or eliminate genetic material within human cells. Gene therapy has shown promise as a means of treating inherited and chronic diseases and degenerative diseases by replacing, modifying or eliminating the genetic material inside human cells [1–3]. Although current methods of gene therapy are encountering many challenges, including unintended actions in places that should not be affected by gene therapy (or transgene therapy), limited types of cells that can be targeted with specificity through gene therapy, and uncertainty in attaining the desired amounts of gene expression, gene therapy has now advanced significantly. Traditional methods of providing stable transgenic expression utilize AAVs as vectors with most promoters being of viral origin and providing some degree of selectivity when driving transgenic expression. However, little is known about the ability to have the right type of promoter to control transgenic expression at the appropriate level. Similarly, while Clustered Regularly Interspaced Short Palindromic Repeats (CRISPR)-mediated genome editing offers strong gene modulation capabilities, it also carries risks such as unintentional genomic alterations and immune responses. Enhancer-based approaches have emerged as a revolutionary solution to these problems, providing specificity for cell types, control over spatial and temporal expression, and flexible gene regulation. Known as non-coding cis-regulatory DNA elements, enhancers play a key role in regulating gene transcription by contextually recruiting transcription factors (TFs) and cofactors. Promoters generally act locally; while enhancers act at long distances. The functional architecture of enhancers is defined by certain histone modifications, such as H3K27ac and H3K4me1, as well as open chromatin accessibility and mediator complexes supporting RNA polymerase II recruitment [4–6]. Recent developments in high-throughput sequencing techniques—such as transposase-accessible chromatin sequencing (ATAC-seq), chromatin immunoprecipitation sequencing (ChIP-seq), and single-cell multi-omics—have enabled the systematic identification and analysis of active enhancers across different cell types and pathological conditions [7, 8].

Achieving precise transgene expression while reducing unintentional ectopic effects is a major difficulty in gene therapy. Using enhancer-based techniques takes advantage of the natural regulatory properties of these components, hence enabling transgene expression customized to particular cell types. Including neural enhancers into gene therapy plans, for example, limits expression to glial cells and neurons, hence improving therapeutic specificity for neurodegenerative diseases like Alzheimer’s and Parkinson’s. Likewise, while microglia-specific enhancers can focus on neuroinflammatory pathways, liver or muscle-specific enhancers can target metabolic and muscular diseases. By combining enhancer components and tissue-specific promoters, gene therapy methods can achieve extraordinary accuracy, hence reducing the possibility of unintentional expression and non-scientific. „off-target effects [9, 10].

Apart from selectivity, the ability to alter gene expression also helps to improve therapeutic outcomes. Too many therapeutic genes can cause cell harm; too little expression might have no clinical benefits. Methods in synthetic biology, such as CRISPR-Cas-mediated enhancer editing and dCas9-based activator/repressor systems, enabling reversible and inducible changes of enhancer activity [11, 12]. This is especially important for complex diseases, in which multiple genes influence the underlying condition. Focusing on disease-associated enhancers enables gene therapy to influence gene regulatory networks rather than a single gene, thereby enabling the recovery of disrupted transcriptional processes. Targeting enhancers that regulate APP (amyloid precursor protein) synthesis influences gene regulatory networks; conversely, manipulating enhancers linked to tumor suppressor genes may hinder glioblastoma development [13, 14].

In vivo gene transfer still relies on AAV-based vectors; adding enhancer components to AAV constructs can improve targeting accuracy. Designed to enable targeted transgene expression in particular neuronal populations, enhancer-engineered AAVs also increase blood-brain barrier permeability for the treatment of central nervous system diseases [15, 16]. Furthermore, thorough functional screening of enhancer libraries helps to find new regulatory sequences with possible therapeutic uses, particularly in diseases with poorly understood genetic pathways [17, 18].

Although they offer advantages, several challenges limit the widespread use of enhancer-driven gene therapy. Enhancer activity is strongly influenced by the specific context, including cell identity, developmental stage, and external factors. Unwanted interactions between promoters and enhancers or improper activation, thus, could cause unusual gene expression, which could have negative consequences. In rapidly proliferating cells, the longevity and consistent function of enhancer components pose greater technological challenges. Delivery effectiveness in bigger animals is still a major issue since systemic administration calls for very exact targeting techniques [19].

Recent developments in synthetic biology, genetics, and computer modeling are addressing these issues and enabling the development of more efficient drugs that rely on enhancers. By outlining regulatory networks with exceptional accuracy, single-cell and multi-omic technologies are improving the identification of enhancers. Engineered synthetic enhancers control gene expression more powerfully than their natural equivalents. Moreover, CRISPR-based enhancer perturbation tools, such as dCas9 activators and repressors, provide scalable and flexible transcriptional control. When combined with other therapeutic methods as RNA therapies and small molecules, strategies focused on enhancers could improve the efficacy of treatments [20, 21].

## Biological roles of enhancers

**Fig. 1:**
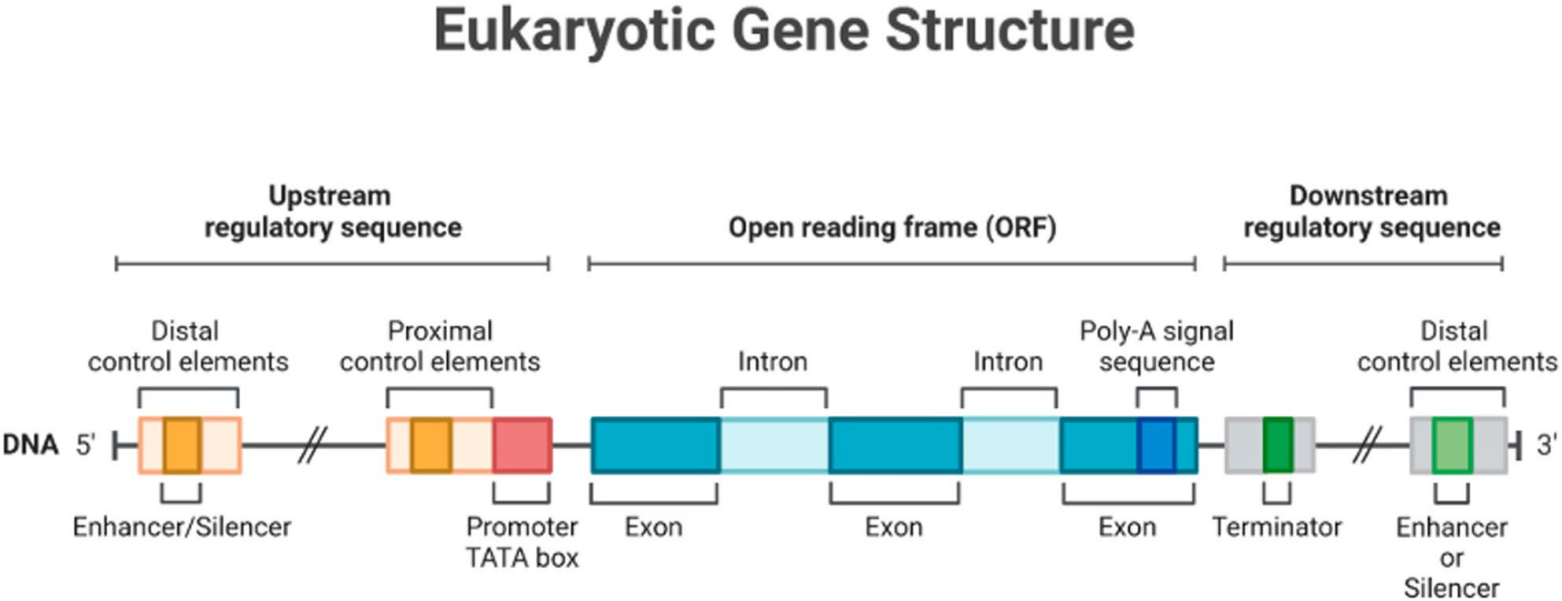
Eukaryotic genes have a modular arrangement of transcriptional regulatory elements and coding sequences, which are collectively responsible for the overall transcriptional output of the gene. Besides the main promoter parts needed to attract the basic transcription tools (transcription factors and RNA polymerase II), there are other regulatory elements far away (called distal regulatory elements or enhancers) that can influence how much a gene is expressed by interacting with these main promoters from a distance. Examples of the types of regulatory elements include enhancers, silencers, and insulators. The effectiveness of these enhancers mainly depends on whether transcription factors can attach to them and how accessible the chromatin is in that area, as well as the epigenetic state of the region. Enhancers can be found before, after, or even inside the non-coding sections of the gene, showing that their location can vary and is important for creating treatments and changing how certain genes are expressed. Exons comprise the protein-coding parts of the gene, while introns are considered non-coding parts that are excised during RNA processing. This diagram provides a conceptual framework for the potential usefulness (or limitation) of enhancer sequences in gene therapy applications

Eukaryotic genes are arranged in a modular style with non-coding components, coding regions, and regulatory sequences. We have amended this sentence as follows: Regulatory enhancers add another layer of specificity to gene regulation, often being located far from the genes they regulate, and they also help regulate gene expression by specifying which genes are expressed. The promoter remains the primary element required for transcription to begin (Fig. 1).

Enhancers are significant because they are not always found right next to where they activate gene expression (as is the case for promoters). While over time they may become more common due to sequence similarity, there is also a large number (many more than promoters) that are comparable to a distance upstream, downstream, or within an intronic region from their target gene(s) that are presently known. It is the chromatin loops alone that bring together enhancer-bound transcription factors (TF’s) and the promoters of target gene(s), enabling the activation of transcription on those genes. The enhancers work in concert with chromatin remodelers and co-activators to regulate how gene expression patterns develop by influencing the recruitment of RNA polymerase II, histone modifications, and DNA accessibility for transcription [22, 23].

### Enhancers in development and cell type-specific gene regulation

The major focus of many scientists studying developmentally regulated genes is on enhancers and their role in controlling gene expression temporally and spatially. Enhancers regulate tissue specification and organ formation by enabling precise control of gene expression in multicellular organisms. In addition, enhancers regulate many genes involved in determining stem cell fate, lineage commitment, and morphogenetic processes during embryogenesis. Enhancers are specific to particular transcription factors and therefore regulate the expression of different genes across different tissues [24]. Newer technologies, such as CRISPR-based enhancer screens and Self-Transcribing Active Regulatory Region Sequencing (STARR-seq), have enabled the identification of cell-specific enhancers that support cell differentiation and development, thereby providing critical tools for researchers to uncover the role of specific enhancers in these processes [25, 26].

Enhancers play an important role in regulating gene expression during embryonic development and throughout life, thereby maintaining tissue homeostasis and cell identity. In particular, enhancers within the immune system regulate the expression of genes involved in antigen presentation, cytokine production, and immune cell proliferation. The controlled activation of enhancers in immune cells allows for the appropriate transcriptional response to pathogens, thereby regulating both the innate and adaptive immune responses [27]. Capture-C and Hi-C are high-throughput techniques for chromatin conformation capture that have provided critical insights into the connections between enhancers and promoters in many types of immune cells. These findings indicate that numerous different regulatory networks exist which regulate immunity.

### Enhancer dysregulation in disease

Alterations to the epigenome and mutations of genetic regions can lead to alterations in enhancer activity that result in incorrect activation (upregulation) or repression (downregulation) of genes, which in turn may lead to the development of a disease state. For example, in many types of cancer, enhancers can be hijacked by oncogenes to promote tumor growth. Specific DNA rearrangements of structural elements adjacent to an enhancer have been demonstrated to create a transcriptional relationship between genes (proto-oncogenes) and enhancers, which contribute to the uncontrolled expression of proto-oncogenes.

Neurodegenerative conditions, such as Alzheimer’s Disease, have altered gene regulatory environments or enhancer dysfunction that result in neuronal function and homeostasis being negatively affected. Altered transcriptional programs due to enhancer dysfunction result in disruption of synaptic plasticity, cellular metabolism, and neuroinflammation, and work towards advancing the disease [28, 29]. Several recent studies of epigenomic profiling and epigenome-wide association studies (EWAS) at a level of resolution that allows researchers to define the genomic regulatory elements (enhancers) associated with neurodegenerative diseases has begun to provide insight for targeting these regulatory elements for therapeutic purposes [30].

### Enhancer-based therapeutic strategies

Enhancers are compelling candidates for precision medicine because they play vital regulatory roles. Especially for diseases brought on by dysregulated gene expression, altering enhancer activity offers a novel strategy for therapeutic intervention. CRISPR-based methodologies, such as CRISPRa (CRISPR activation) and CRISPRi (CRISPR interference), have enabled precise modulation of enhancer activity to promote or suppress gene expression in disease-related contexts. Under study to control enhancer activity in cancer and inflammatory illnesses are small-molecule epigenetic modifiers, including bromodomain inhibitors [29, 31].

When compared to expressions driven via promoters alone, using enhancers together with the molecular information contained in an enhancer provides enhanced context sensitivity since they can bring together endogenous regulatory cues from cellular processes close to them. Nevertheless, enhancer activity does not guarantee physiological measurements will be congruent with whatever has been expressed via the use of the actual enhancer and can also vary widely across cell types, stages of development, and diseases present. As a result, enhancer “leakage” (ectopic expression) remains a consideration when evaluating the cellular context and must be viewed in a manner that distinguishes ectopic gene expression from actual off-target effects found in the act of genome editing. Rigorous validation (ideally at single-cell resolution) is, therefore, needed to show enhancer-dependent specificity as well as to assess the reproducibility and longevity of enhancer-driven transcriptional output.

Through tissue-specific and condition-dependent gene regulation, enhancer-based approaches offer distinct (Table 1). Innovative ideas, including single-cell enhancer profiling, high-resolution chromatin interaction mapping, and AI-driven enhancer prediction models, will increase enhancer-targeted therapies as our knowledge of enhancer biology develops [32, 33]. More studies may be needed that employ enhancer-centric approaches to gene therapies that require precise gene regulation in humans. Enhancers are critical for regulating genes at all levels, including developmental processes, immune function, and cell homeostasis. Aberrant enhancer activity has been shown to contribute to many diseases; therefore, future studies focusing specifically on enhancers as a viable therapeutic target are warranted. Additionally, recent technological advances in both epigenomics and genome editing have enabled the development of enhancer-targeted therapies to treat various cancers and neurodegenerative diseases, thereby expanding our ability to provide precision medicine for a variety of disorders.

**Table 1:** Compare two gene therapy strategies: enhancer-based and promoter-based techniques. Enhancer-based methods use DNA fragments called enhancers to precisely control gene expression with respect to cell type and flexible regulation. Still, they face challenges, including the need for precise enhancer targeting and the difficulty of linking enhancers to particular genes. On the other hand, promoter-based methods emphasize the promoter areas of genes, which are crucial for beginning transcription. Although they lack the flexibility of enhancer-based approaches and could cause off-target effects because of their broad impact on gene networks, these well-known and simple processes are not as adaptable

Strategy	Strengths	Weaknesses
Enhancer-based	- Cell-type specificity - More flexible regulation	- Difficult to assign enhancers to target genes - Requires precise enhancer targeting
Promoter-based	- Simplicity - Well-established	- Less flexibility - Potential for off-target effects

### Enhancers and cis-regulatory elements

Enhancers are essential for development and differentiation, regulating genes involved in patterning the body, establishing tissue identity, and determining cell fate during embryonic development. They regulate which genes are activated in the right cell types and at the appropriate times during the process of embryonic development. Some enhancers are selectively utilized in certain types of cells for controlling the expression of genes critical for immune responses, thereby limiting the unnecessary activation of genes in non-immune cells [31, 34].

Though prominent cis-regulatory elements, enhancers are not the only factors influencing gene expression. There are several other regulatory areas in the genome, including insulators, silencers, and promoters. Promoters mostly connect with RNA polymerase and different transcription factors next to the transcription start site of a gene. While silencers lower gene expression by drawing repressive transcription factors, insulators prevent enhancers from interacting with non-target genes [34].

Accurate regulation of gene expression across multiple biological systems depends on the coordinated activities of enhancers, promoters, insulators, and silencers. Together, these four types of elements balance multiple aspects of cellular homeostasis, promoting gene expression via enhancers while limiting latent gene activation via insulators and silencers. Disruption of these regulatory pathways can lead to serious developmental abnormalities, neurological disease, and cancer. Enhancer element mutations are often associated with oncogene activation and subsequent tumor growth. Changes in enhancer function associated with neurological diseases can also lead to dysregulation of genes involved in synaptic function and subsequently lead to disorders like Alzheimer’s. ^35,36^.

Since enhancers play an essential role in regulating Gene Expression, enhancing gene expression is becoming an increasingly important target for developing therapeutics. Enhancers are key therapeutic targets in conditions such as cancer and autoimmunity, as blocking pathogenic enhancers can decrease the production of harmful genes responsible for tumor formation or inflammation. Conversely, activating specific enhancers would facilitate treatment of conditions associated with underexpression of therapeutic genes (e.g., Duchenne Muscular Dystrophy, cystic fibrosis) [35].

Because of advances in CRISPR/Cas9 and other gene-editing technologies, we can now regulate enhancer activity and develop new targeted treatments. By altering enhancer function, we may be able to improve treatment efficacy, enhance treatment specificity, and reduce adverse effects associated with certain inherited disorders [36]. Because they modulate gene activity during development and disease, enhancers may be important targets for developing new therapeutic strategies. As gene-editing technologies advance, the potential for enhancer-based precision medicine will expand, providing new opportunities to develop therapies for a variety of diseases.

### Enhancer structure and roles in transcriptional regulation

**Fig. 2:**
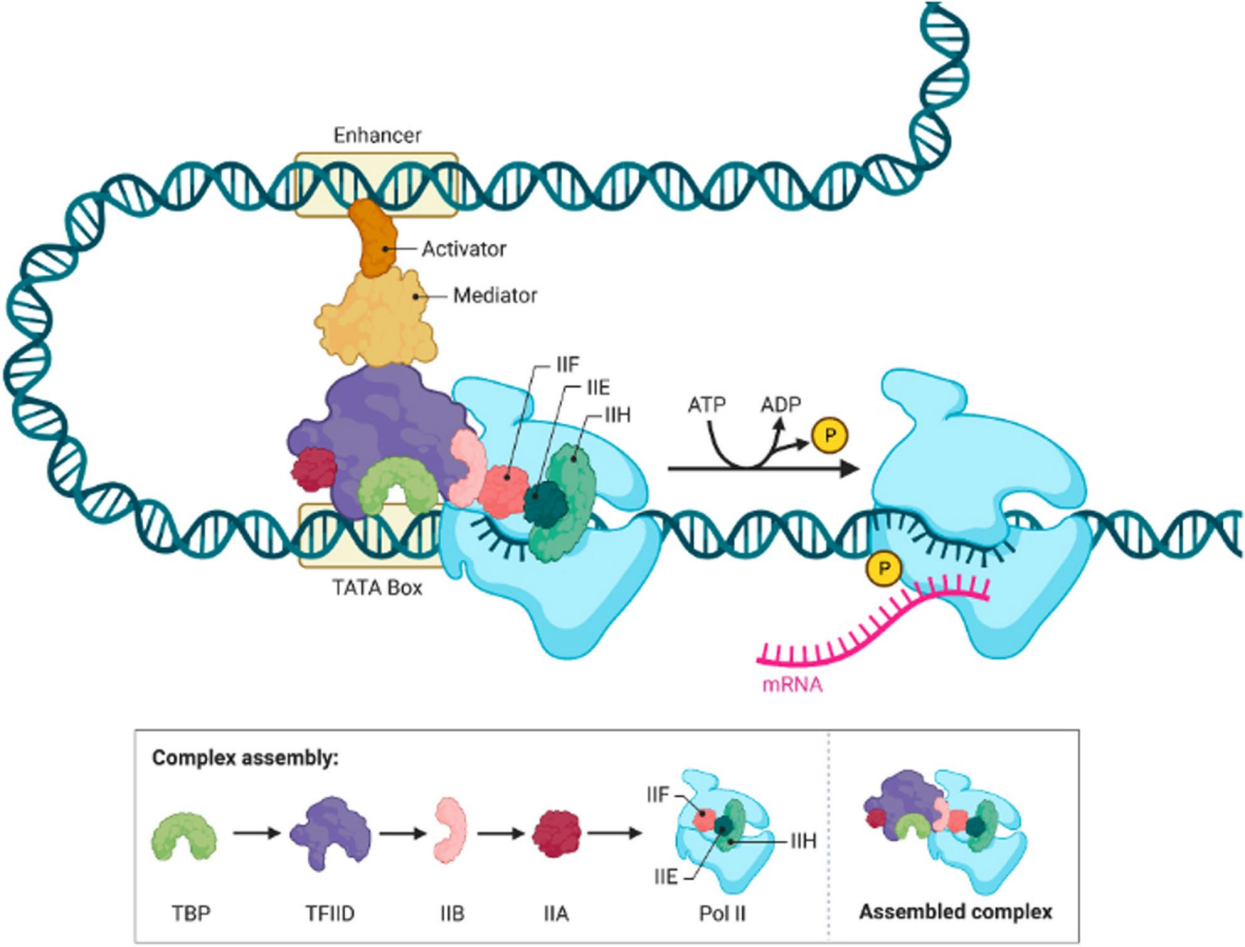
The process of transcription initiation occurs in eukaryotes. At the time that transcription starts, a pre-initiating complex, or the "PIC," is formed at every core promoter. Together with the distal regulatory elements, the PIC are functionally integrated into one another. When enhancer-bound transcription activators (transcription factors that increase transcription) bind to mediator-type proteins and form a template, transcription factors bound to the core promoter communicate with enhancer-bound transcription factors via chromatin-looping. Transcription begins by putting together a PIC in steps; the first step is when the TATA-binding protein (TBP) attaches to the core promoter as part of the TFIID protein complex. After that, additional general transcription factors (TFIIA, TFIIB, TFIIF, and TFIIE) and RNA polymerase II are recruited to the core promoter. The phosphorylation of the CTD on RNA polymerase II by the TFIIH protein complex, which uses ATP as an energy source, is the last step of the transition of transcription from initiation to elongation. The following schematic shows how enhancer activity, availability of transcription factors, and promoter structure all together determine the overall amount of transcription, thus confirming that enhancer-promoter interactions are context dependent and have implications for regulating endogenous gene expression and for enhancing therapeutic strategies [38]

Enhancers work through several mechanisms; however, transcription factors are very important for mediating this interaction (among all ways that enhancer elements interact with their target). There are many ways in which fusion genes can occur as a result of genomic reconstruction, including but not limited to, DNA recombination and chromosomal translocations that reposition two separate genes together to produce a new, mutated gene - a hybrid of two separate forms of DNA (Fig. 2). The three-dimensional shape of DNA allows physical contact between promoters and enhancers, hence placing the enhancer close to the promoter area of the gene. Chromatin looping and the activity of architectural proteins like CCCTC-binding factor (CTCF) and cohesin help to support this spatial reconfiguration by strengthening these links [37].

Transcription factors and epigenetic modifications, including histone acetylation and DNA methylation, influence enhancer activity. Often, histone acetylation is connected to an open chromatin structure that helps transcription factors attach, hence increasing gene activation. On the other hand, gene suppression and lower enhancer activity are often linked with DNA methylation [39].

### Emerging technologies for enhancer research

Recent technology developments have greatly improved our ability to explore enhancer biology. Enhancer screening has been revolutionized by high-throughput techniques such Hi-C, CRISPRa/i screens, and STARR-seq, hence enabling the discovery and functional validation of enhancers all over the genome. By including enhancer fragments into reporter assays, STARR-seq enables the high-throughput measurement of enhancer activity. At the same time, CRISPRa/i screens offer the exact activation or suppression of particular enhancers to assess their functional impact on gene expression. A chromatin conformation capture technique, Hi-C clarifies long-range chromatin interactions and helps to trace enhancer-promoter links across the whole genome. These tools offer unmatched understanding of enhancer function and its relevance in gene control [40, 41].

### Comparative analysis of enhancer- vs. promoter-based gene regulation

While both enhancers and promoters function as important elements controlling gene expression, they perform distinct roles and can be used differently in developing therapies. Most often, promoters work by providing more direct control of transcription at the transcription start site through the binding of transcription factors to promoters. Enhancers, on the other hand, are frequently involved in tissues/stage-specific regulation of gene expression, and they act at a distance from the transcription start site. The inherent flexibility associated with these regulatory mechanisms allows enhancers to be involved in multiple regulatory systems for the control of gene expression throughout both normal development and disease [42].

The use of enhancer-based methods has several benefits compared to promoter-driven methods in therapy. In conditions where aberrantly regulated enhancers result in abnormal gene expression, the use of enhancers as targets will allow for a better ability to modulate gene expression. In contrast, in conditions characterized by mutations in the promoter region or the requirement for sustained and/or robust gene expression, promotor driven methods would be preferable [43]. In order to develop effective enhancer-driven gene therapies, it is necessary to have a detailed mapping of enhancer-promoter interactions along with an understanding of the cell type specificity of those enhancers. Strategies that target enhancers will increase the likelihood of activating the desired enhancer in the correct cell types through the use of tissue specific delivery systems such as modified AAV capsids [44].

With the arrival of new technologies and methods for enhancer targeting will have a major influence on gene therapy in that there will be much greater precision and specificity in treating genetic disorders. Tissue-specific gene expression is a vital part of the identity and function of a cell and allows for specialised function at various levels of organ and tissue within a living organism. In the complex network of genetic and epigenetic regulations for gene expression, enhancers are essential [45].

A key characteristic of all enhancers is that they can function as inactive and active according to the type of cell where they reside. Individual organs have unique transcription factor environments that bind to these enhancer regions which either activate or repress gene expression. Typically, tissue-specific enhancers can be located within specific genomic regions defined by specific patterns of histone modifications (acetylation and/or methylation) and demonstrate an open chromatin structure that facilitates transcriptional activation. On the other hand, areas of compression defined by DNA or histone methylation usually correspond with repressed enhancers [46].

Activation of specific enhancer tissue will be done in and will require several levels of regulation that are highly regulated. The first layer of regulation will be transcription factors that are needed to initially interact with enhancers; individual transcription factors will be specific to specific cell types and will recruit/guide co-activators and other regulatory proteins to eventually initiate transcription. Some transcription factors may be present prior to cell development and other transcription factors may be activated by exogenous signals such as hormones or growth factors. Pluripotent stem cells show a particular spectrum of transcription factors activating enhancers controlling genes necessary for cell differentiation throughout early development. As cells differentiate into specialized lineages, they express transcription factors that fit the particular functions of the tissue or organ [47]. By dynamically controlling enhancer activity, these epigenetic changes guarantee that genes are expressed only when required. While those for muscle-specific genes get more accessible and active throughout muscle growth, enhancers for non-muscle genes stay repressed [48]. Chromatin looping allows long-range interactions by helping enhancer and promoter areas to be close to one another [49]. This capacity governs the coordination of gene expression throughout the genome, allowing enhancers to simultaneously influence gene control at many genomic sites.

**Fig. 3:**
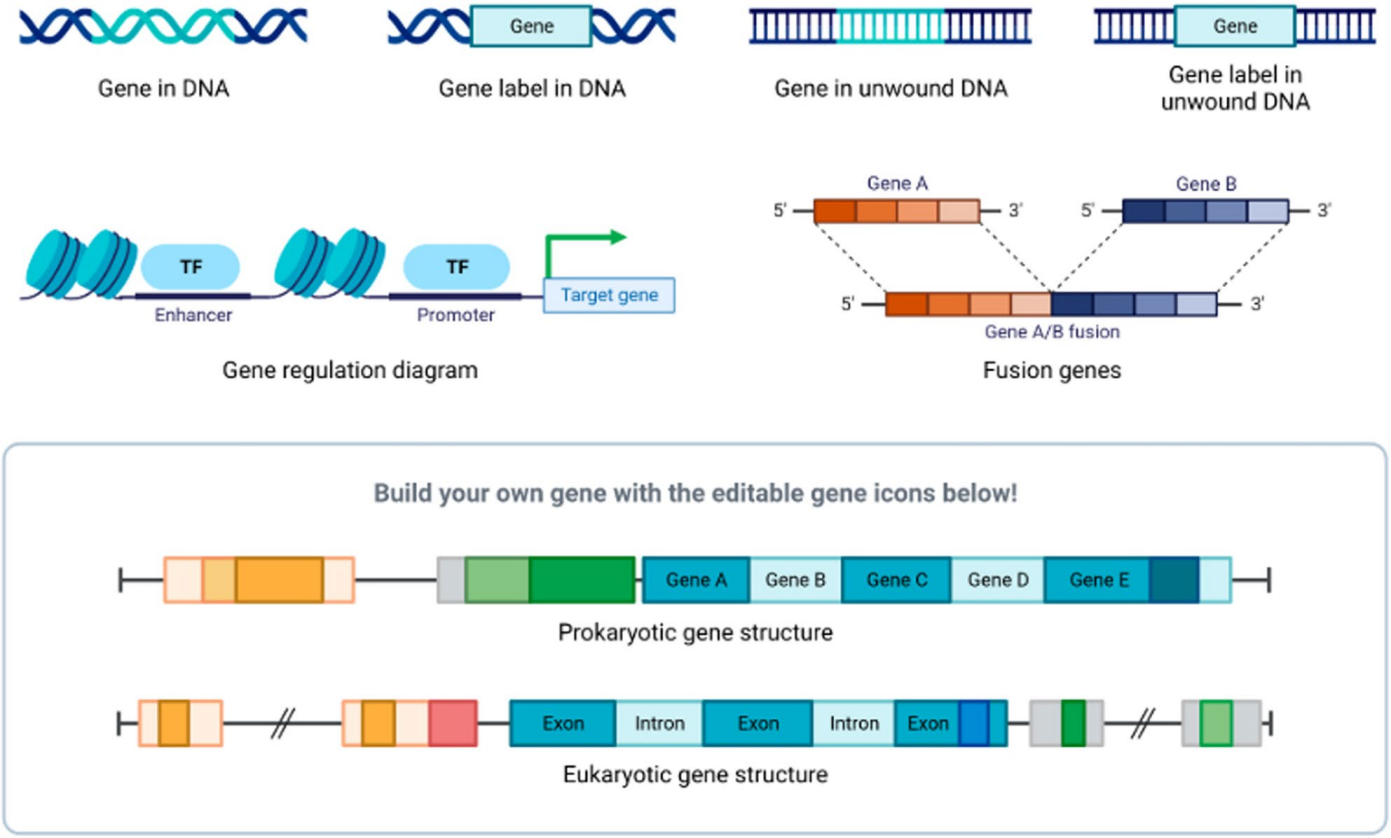
Gene regulation and structure. The upper side demonstrates where the two components (enhancers and promoters) that help gene regulation are located. They interact through chromatin to combine their effect on regulating a given gene (the target gene). Enhancers and promoters work together in an interactive fashion with transcription factors to coordinate the transcription (the process of turning the DNA into RNA) from enhancer to promoter as well as providing the signals to begin transcription and allowing transcription to occur from the gene. In addition to being able to coordinate transcription from enhancers to promoters, enhancer-promoter interactions also allow for additional ways of starting transcription (providing the corresponding signals to other proteins for transcription to occur). Importantly, enhancer-promoter interactions are not only dependent on the levels of transcription factors, chromatin accessibility, and the genomic context of the enhancer and promoter, but also on the stochastic (non-deterministic) interaction of the enhancer and the promoter (2:1), e.g., a given transcription factor can bind to more than one enhancer, a given enhancer can be bound by more than one transcription factor, and they can act at the same time on the same enhancer (and vice versa). The lower diagram depicts that using two distinct genes, the new chimeric gene results from the chromosomal rearrangement (via recombination/translocation). This figure provides an example of how genomic rearrangements can alter both the regulation and the coding structure of genes. However, this figure is not given as an illustration of the gene therapy that is aimed at enhancing the gene as a result of the enhancer-driven regulatory designs that have occurred to create the new chimeric gene. Through a process called chromatin looping, enhancers and promoters become physically close to one another by utilizing transcription factors, co-activator complexes, and the general transcriptional machinery working together. When at this location, enhancers also allow transcription factors bound to them to activate RNA polymerase II and general transcription factors bound to the promoter, thereby increasing the probability that transcription will start, which will then lead to the synthesis of RNA. A schematic representation is given that indicates how the three-dimensional shape of the chromatin itself alters the communication that occurs between enhancers and promoters depending on their distal, regulatory role. Factors such as chromatin accessibility, availability of transcription factors and/or regulatory factors, and the state of the cell all impact transcriptional activation. This overall regulatory logic provides the basis for many of the methods that use enhancers as a means of regulating transcription, and also illustrates important limitations associated with their application in the therapeutic setting [52]

Enhancers greatly affect how cells go from pluripotent stem cells to fully differentiated, tissue-specific cells. Enhancers control genes deciding cell destiny choices throughout early development. Enhancers get more and more specific to tissue types as development progresses; so, specialized activities depend on gene activation. Whereas enhancers for non-neuronal genes are suppressed, those for neuron-specific genes such as those controlling ion channels and neurotrophic factors are activated during brain development (Fig. 3). During muscle development, muscle-specific enhancers turn on genes linked to contraction and growth [50, 51].

Apart from their role in growth, enhancers are crucial for maintaining cell identity all through an organism’s lifetime. Once a cell has taken a certain sort, such as a muscle cell or neuron, enhancers keep gene expressions defining its identity. In this way, tissues and organs have to perform constantly. In neurons, enhancers help to generate the ion channels and neurotransmitter receptors required for signaling; in muscle cells, they maintain the expression of genes necessary for contraction and repair [53].

### Synthetic biology and enhancer-driven gene therapy

A key aspect of synthetic biology and gene therapy, gene expression is precisely regulated by enhancers, which are hence quite useful tools. Synthetic biology focuses on the design and building of new biological components, technologies, and systems. In this regard, enhancers are absolutely vital since they increase gene expression and help to create strong and manageable gene control systems. In synthetic biology, the main benefit of enhancers is their capacity to greatly increase gene transcription when located upstream of a target gene, beyond the regulatory power of promoters alone [54].

Furthermore, enhancers can be designed to respond to certain stimuli, including environmental cues, therefore enabling dynamic control of gene expression. In systems requiring precise control of gene expression, such as in engineered microbial systems or mammalian gene treatments, this capacity is very beneficial. Metabolic engineering improves microbial cells to create desired compounds or biofuels. Including enhancer sequences encouraging the synthesis of essential enzymes will increase the effectiveness of these systems in generating the needed outputs [55].

Used in industrial biotechnology, synthetic promoters and enhancers speed up the production of proteins or biologics, which is absolutely necessary for large-scale manufacturing operations [56]. Mammalian synthetic biology relies on enhancers for tissue-specific gene expression in gene therapy applications. The use of liver-specific enhancers can help reduce off-target effects and improve therapeutic outcomes for gene therapies that deliver therapeutic proteins to the liver [57].

Synthetic gene circuits are created by mixing together various enhancers that work together to modulate how genes are expressed depending on different signal inputs and the corresponding combination of more than 1 enhancer plus other regulatory elements. The basic method of this process is to create more complex living systems that can perform useful functions such as detecting poisons, controlling metabolism, or regulating apoptosis [58]. In order for these gene circuits to operate as designed in various in vivo situations, coordination of enhancer activation is essential for proper function. Recent innovations in synthetic biology allow for the use of synthetic enhancer libraries. These libraries contain a wide variety of different enhancer sequence types. These libraries allow scientists to test each enhancer’s ability to modulate gene expression across different conditions while overcoming the limitations found with naturally occurring enhancers (e.g., tissue selectivity and regulatory restrictions).These libraries provide a fast mechanism for maximizing gene expression in manufactured systems [59]. Additionally, synthetic enhancers that can respond to external signals (either from an environment or an artificial signal) may allow for greater flexibility and efficiency when using gene expression systems [60].

With the ability to systematically discover enhancers at genome scale as well as use many high-throughput techniques to do so, enhancer-based approaches have fundamentally transformed the field of re-sequencing enhancers, including STARR-sequencing and CRISPRa/i screens [61, 62]. These technologies provide possibilities to treat illnesses such as Alzheimer’s disease or cancer. These technologies permit you to create enhancer maps with higher resolution than previously possible and use this information to increase the efficiency of using gene therapy to treat individuals with these conditions.

Increased opportunities for synthetic biology’s ability to manipulate gene expression through high-throughput enhancer screening methods may lead to new therapeutic uses, such as the development of gene therapy using specific targeting methods for different tissues and gene expression control in disease models. It is generally understood that when compared to traditional promoter-driven gene therapies, enhancer-based gene therapies provide a greater degree of accuracy in directing gene expression to a particular cell type when combined with other technologies (e.g., CRISPR-Cas9, chromatin conformation capture) [63, 64].

All biological processes that maintain cellular identity, regulate genes and gene expression, and ensure tissue-specific gene activation are reliant on the action of enhancers. Enhancers derive their functionality from the influence of transcriptional regulatory factors and epigenetic modifications. Enhancers have the property of being very dynamic, thus enabling the precise regulation of gene expression needed for optimal functioning of the cell. The source of an extremely high degree of control of gene expression for synthetic biology and gene therapy projects is the use of enhancers, providing the opportunity to create tissue-specific gene therapies and ultimately synthesize gene circuits. The increased use of high-throughput screening methods in conjunction with new and innovative gene editing technologies is firmly establishing enhancers as key components in the therapeutic area and providing new opportunities for treating more complex diseases.

Enhancer screening is an integral aspect of the molecular biology discipline that provides new insight into how genes are regulated and, through the use of enhancers, allows targeted therapeutics to be used in conjunction with enhancers. Developing embryos depend upon a number of different types of cis-regulatory elements for the temporal and spatial regulation of gene expression as they develop. The main means by which enhancer activity is identified and validated is through profiling methods (such as chromatin accessibility assays such as ATAC-seq or DNase-seq) using advances in genetic and molecular biology technologies (Jones et al. 2016 & Hurst). These investigations help to find open chromatin regions linked to active enhancers. Furthermore, histone modification chromatin immunoprecipitation combined with sequencing (ChIP-seq) for H3K27ac and H3K4me1 helps to find active enhancer regions. Furthermore, lower DNA methylation levels in some sites could suggest enhancer activation. Enhancers can produce non-coding RNAs called enhancer RNAs (eRNAs), which RNA sequencing (RNA-seq) can identify as indicators of active enhancers [65, 66].

Significantly improving our ability to find enhancers on an unmatched scale, high-throughput techniques such Massively Parallel Reporter Assays (MPRAs) allow the simultaneous assessment of thousands of sequences for enhancer activity. Providing knowledge on the control of gene expression at the cellular level, single-cell ATAC-seq and multi-omics techniques have proven important in finding cell-type-specific enhancers. These methods enable high-resolution studies that provide a better understanding of enhancer activity in certain cell types and pathological contexts [67].

In gene therapy, enhancers are quite important for therapeutic uses. Their ability to precisely control gene expression makes them vital in the development of more efficient and focused gene therapies. Enhancers play a crucial role in the regulation of lineage- specific gene expression through development; therefore, the identification of enhancers as regulatory components contribute to our understanding of stem cell differentiation and general developmental biology. There are many disease states (such as malignancies) in which enhancers have been shown to be hijacked—enhancers that are not normally active have been inappropriately activated to become pathological by facilitating the improper activation of proto-oncogenes or oncogenes, which create opportunities for developing new therapeutic targets and/or diagnostic markers [68]. Neurodegenerative diseases like Alzheimer’s and Parkinson’s have also been associated with enhancer molecules. The study of enhancers and their role in various diseases may provide valuable information for future therapies [69].

### Creative approaches and technologies focused on enhancers

New technologies are completely changing how we examine and manipulate enhancer elements. High-throughput methods such as STARR-seq, CRISPRa/i/screenings, and Hi-C allow for the identification and manipulation of enhancers across the entire genome.The STARR-seq technique utilizes high-throughput methods to evaluate whether enhancers are capable of activating the expression of downstream genes. The CRISPRa/i/screenings method is a potent tool applied in functional genomics that allows for either the activation or inactivation of selected enhancer sequences. This approach is clearly advantageous in allowing researchers to test and examine the functional action of specific enhancer elements.

In addition, researchers can map long-distance enhancer-promoter interactions utilizing Hi-C and various chromatin conformation capture techniques. This information will help researchers understand how enhancer elements influence the expression of nearby genes [70, 71].

Notwithstanding these changes, the distribution of enhancers to their appropriate target genes still presents a major challenge. Dynamic and context-dependent enhancer-promoter interactions make finding target genes with their long-range properties more difficult. Cell type and the surrounding environment influence the activity of enhancers, hence complicating the generalization of results across several cellular settings. Moreover, a thorough analysis of large datasets generated by high-throughput screenings calls for sophisticated computing methods. A complete understanding of enhancer activity and an enhancement of the precision of therapeutic uses depend on the integration of proteomics, transcriptomics, and epigenomics [64, 72]. Emerging artificial intelligence (AI) systems predict enhancer activity and find gene targets depending on sequence and epigenomic features, therefore offering a new approach to these problems [73].

### Enhancers in cellular identity and transcriptional memory

**Fig. 4:**
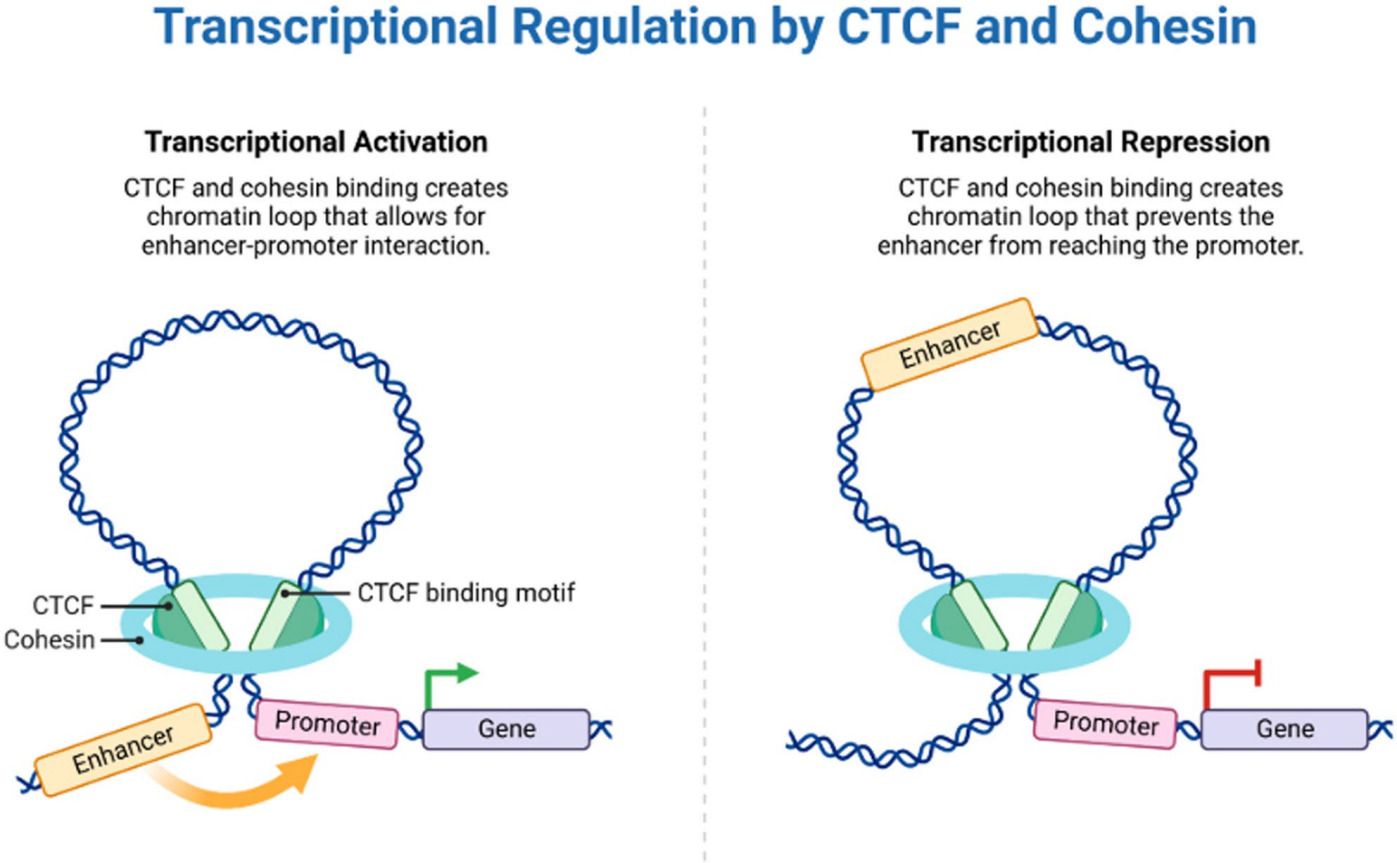
Transcription activation. The left panel shows how transcriptional activation happens through chromatin looping, where cohesin and CTCF help create or maintain a 3D structure in the genome that brings an enhancer close to a target promoter. This proximity can increase the likelihood of productive enhancer–promoter communication and transcriptional activation, depending on local chromatin accessibility, transcription factor occupancy, and cellular context. The right panel shows a setup where chromatin looping helps stop transcription by keeping enhancers away from promoters or by protecting promoters from distant regulatory signals. In this context, CTCF-bound boundary elements and cohesin-mediated loops can limit enhancer influence, thereby reducing transcriptional activity. The CTCF binding motif is shown to highlight sequence-specific architectural anchoring points within the genome. Together, these schematics illustrate how three-dimensional genome organization shapes gene regulation by modulating the probability of enhancer–promoter interactions rather than enforcing deterministic activation or repression. This context-dependent architectural control is a central consideration for enhancer-informed regulatory design and endogenous enhancer modulation strategies [75]

Gene expression is controlled and transcriptional memory preserved by enhancers, which are hence vital. This phenomenon refers to the ability of cells to “remember” previous transcriptional states, hence enabling the rapid reactivation of genes in response to ongoing stimulation. Cellular differentiation, immunological reactions, and stress adaptation all depend on transcriptional memory. Enhancers promote transcriptional memory by means of many ways. Linked to active enhancers, histone changes such as H3K4me1 and H3K27ac work as molecular “bookmarks” that prepare genes for reactivation. Even during transcriptional pauses, these changes, together with the production of eRNAs, maintain chromatin accessibility and encourage long-range enhancer-promoter interactions. This dynamic process lets the cell preserve cellular identity across time and react more effectively to environmental changes [74] (Fig. 4).

Transcriptional memory affects several biological processes. Enhancers in immune cells are critical for the immediate reactivation of inflammatory genes when exposed to a pathogen [76]. Enhancers also provide for rapid reactivation of genes involved in response to stress (i.e., heat shock and oxidative stress) that allows cellular adaptation during times of change. Furthermore, enhancers enable neuronal transcriptional memory, hence supporting memory development and learning activities. Dysregulation of enhancer activity could lead to aberrant gene expression, usually connected to diseases including cancer and treatment resistance [77].

### Mechanisms of enhancers and dynamics of chromatin

The 3D architecture of chromatin is critical for how enhancers function. Interactions between enhancers and promoters occur from chromatin looping (mediated by Cohesin, CTCF) and result in transcription factors and co-activator access at the promoter for proper activation of gene expression upon loop formation. Nonetheless, when the chromatin loop is compromised, transcription is inhibited. The dynamic nature of this chromatin conformation is crucial, as its regulation plays a vital role in sustaining gene expression patterns that are important for cellular function and differentiation [78].

Techniques for mapping chromatin interactions, like Hi-C and Capture-C, provide a more profound insight into enhancer-promoter interactions, which tend to be long-range and dynamic. Understanding how enhancers function in gene regulation during complex disease processes will benefit significantly from knowledge of these techniques. This is especially true when considering that chromatin structure can dramatically change the way enhancers function. Mutations in either CTCF or Cohesin genes can affect the looping activity between enhancers and promoters, which in turn affects the regulation of the genes involved in both cancers and abnormal development [79].

### Epigenetic control of enhancers

Enhancer functionality is a regulated activity through epigenetic modifications that affect chromatin architecture and the accessibility of specific genes. Enhancer activity is determined by two major classes of histone modification: acetylation and methylation. These classes of modifications allow one to differentiate whether an enhancer is active or inactive.

**Fig. 5:**
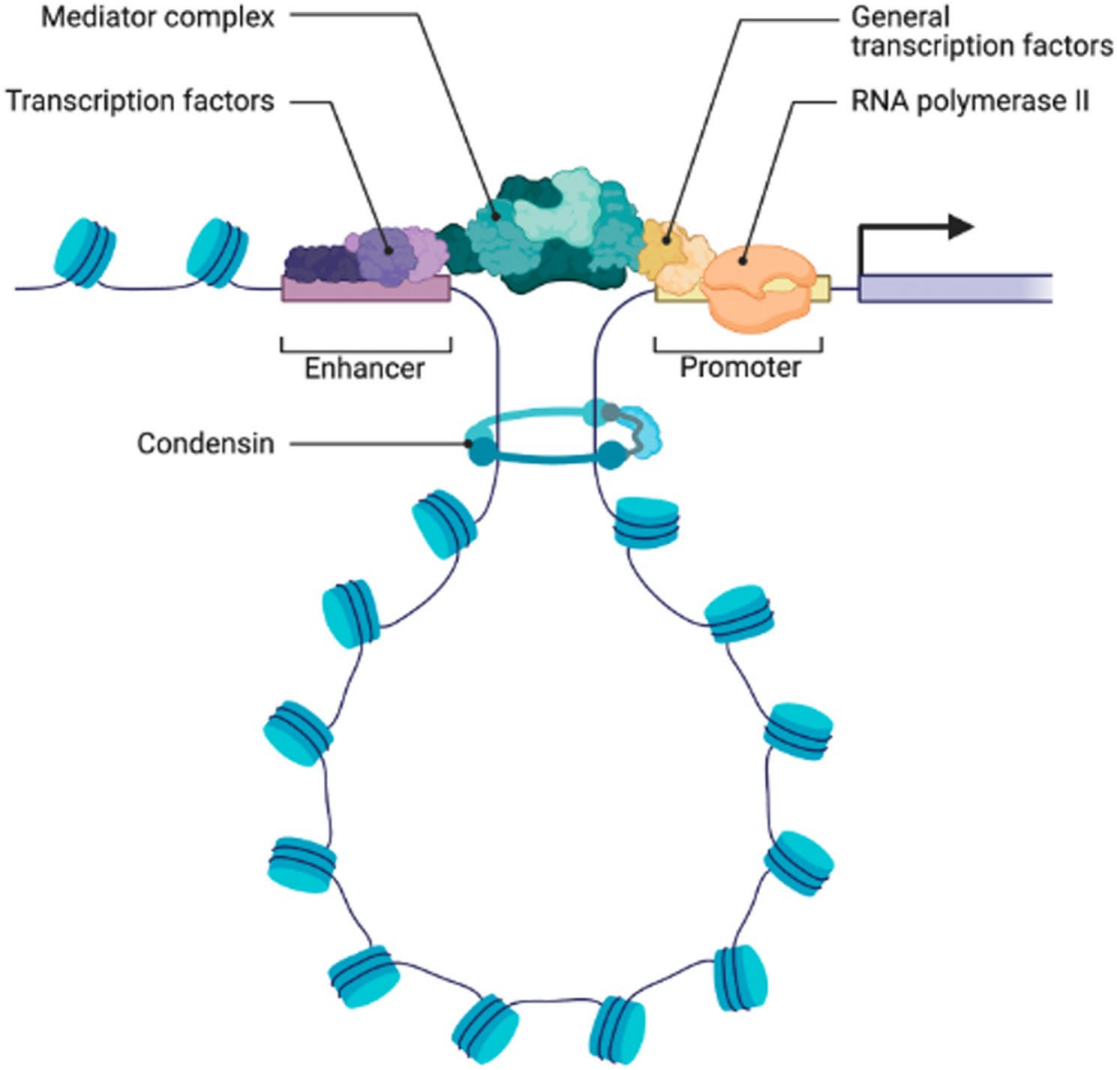
The process involves the interaction between enhancers and promoters through chromatin looping. Through a process called chromatin looping, enhancers and promoters become physically close to one another by utilizing transcription factors, co-activator complexes, and the general transcriptional machinery working together. When at this location, enhancers also allow transcription factors bound to them to activate RNA polymerase II and general transcription factors bound to the promoter, thereby increasing the probability that transcription will start, which will then lead to the synthesis of RNA. A schematic representation is given that indicates how the three-dimensional shape of the chromatin itself alters the communication that occurs between enhancers and promoters depending on their distal, regulatory role. Factors such as chromatin accessibility, availability of transcription factors and/or regulatory factors, and the state of the cell all impact transcriptional activation. This overall regulatory logic provides the basis for many of the methods that use enhancers as a means of regulating transcription, and also illustrates important limitations associated with their application in the therapeutic setting

For example, an active enhancer usually has acetylated histones, which open up the chromatin structure, allowing transcription factors to bind. In contrast, a repressed enhancer usually has methylated histones that compact the chromatin structure, making it difficult or impossible for transcription factors to access [80] (Fig. 5).

DNA methylation plays a crucial role in the regulation of enhancers. DNA methylation generally inhibits enhancer activity by blocking the attachment of transcription factors. In some situations where either development is happening or where there has been disease present, and with those specific situations, there is evidence that when someone develops DNA methylations, that is associated with the activation of an enhancer as well, proving that there are subtleties to epigenetic regulations and how they may vary depending on the environment or context [81].

Non-coding RNAs, including long non-coding RNAs (lncRNAs), play a crucial role in modulating enhancer activity through their interactions with chromatin-modifying enzymes, which can either activate or repress enhancer function. These mechanisms play a crucial role in preserving cellular identity throughout differentiation and development, and they are also significant in the context of disease, where changes in enhancer function may result in abnormal gene expression patterns [78].

## Advances in enhancer-based treatment

### General overview

Dysregulation of enhancer activity is associated with various diseases, including cancer, neurological disorders, and autoimmune diseases. Compared to traditional promoter-centric approaches, enhancer-centric strategies provide significant advantages in gene therapy and precision medicine. Though often used to control gene expression, promoters usually lack the cell-type specificity and exact modulation provided by enhancers [82].

Enhancer research is increasingly important in gene therapy, as researchers can precisely modulate enhancer activity to regulate gene expression. Multi-omics data offer an opportunity to identify enhancers involved in drug resistance or oncogene activation, providing valuable insights into how to develop therapies that modulate these enhancers. Additionally, synthetic enhancers are being developed through multi-omics data to enhance gene regulation in therapeutic contexts, especially for age-related diseases or neurodegenerative disorders like AD [83].

Enhancer-based strategies offer numerous advantages over conventional promoter-based methods in gene therapy. In contrast to promoters that facilitate constant gene expression, enhancers provide a more refined and specific regulation of gene expression tailored to different cell types. Focusing on enhancers enables targeted activation or repression of genes, thereby minimizing off-target effects and improving therapeutic outcomes. A comparative table (Table 1) outlines the advantages and disadvantages of enhancer-based versus promoter-based gene regulation strategies in gene therapy.

Targeting enhancers is essential for regulating genes specific to particular cell types. Recent developments in CRISPR-based technologies, including CRISPR/Cas9 for enhancer knockout and CRISPRa/i for enhancer activation/inhibition, enable precise control over enhancer activity in therapeutic applications. These technologies allow researchers to alter enhancers with remarkable precision, presenting thrilling possibilities for investigating gene regulation and disease mechanisms [84].

The dynamic properties of enhancers make them an interesting target for therapeutic intervention. A variety of strategies for regulating enhancer activity, either by increasing or decreasing the activity of selected enhancers, can create new molecules that may potentially provide therapeutic options for neurodegenerative diseases. For instance, the use of gene-editing technologies or small molecules to restore the functional activity of neuroprotective enhancers may slow or reverse the clinical progression of the disease. In addition, new technologies for developing targeted delivery mechanisms using adeno-associated viruses (AAV) may enable precise regulation of enhancer activity in specific brain regions or cell types, thereby providing a more targeted therapeutic approach [85]. The growth of single-cell transcriptomics and epigenetic profiling provides researchers with access to enhancers that exhibit abnormal activity in neurodegenerative diseases. This helps us understand how enhancers contribute to disease pathways, enabling us to identify new biomarkers for therapy, disease progression, and diagnostics [86].

To enhance the effectiveness of enhancer-based drugs, it is imperative to consider how the drug is delivered and the potential for off-target effects. Viruses such as AAVs (adeno-associated viruses) have been used as viral vectors to deliver enhancers and therapeutic genes to their targets (such as microglia, astrocytes, and brain neurons). The packaging capacity for AAVs is ~ 4.7 kb, which restricts the amount of genetic information they can carry. However, even though the payloads of AAV vectors are small, enhancer sequences (if present) will allow for an increase in the level of gene expression from AAV vectors, allowing for enhanced AAV-based gene therapies [87].

However, the safety and effectiveness of enhancer-based therapy are crucial because enhancer dysregulation may activate non-specific genes through off-target effects, thereby promoting oncogenesis and immune responses. Enhancer-based therapies can be used with tissue-specific enhancers or via direct delivery to limit activation of ectopic enhancers; however, long-term off-target effects must also be considered with enhancer-directed therapies, particularly in cancer and Neurodegenerative diseases [88, 89].

### Enhancer-based therapy for neurodegenerative diseases

Multiple clinical studies on neurodegeneration have used AAV vectors to deliver NGF, BDNF, and GDNF and have been cited in discussions of transcriptional targeting. Unfortunately, these studies have employed traditional gene-replacement strategies driven by promoters and have not considered enhancement-based regulatory mechanisms. These studies, however, are valuable for informing the route of delivery, safety, and therapeutic efficacy of gene delivery to the CNS, but they cannot be considered clinical evidence for enhancer-based regulation. As a result, these studies are treated as contextual examples of gene therapy in this review, as opposed to examples of enhancer-based therapeutic design.

Several studies highlight the link between epigenetic changes at enhancer sites and neurodegenerative diseases. Changes in enhancer-mediated gene expression in Huntington’s disease have been connected to the abnormal growth of the huntingtin gene, which produces dangerous protein fragments. Extensive epigenetic changes in AD, including DNA methylation and histone modifications at enhancers, have been shown to influence immune function and neuronal gene expression, thereby exacerbating disease progression. Moreover, neurodegenerative diseases often target specific cell types, such as neurons, astrocytes, and microglia. The preservation of the identity and function of these cellular subtypes depends on enhancers. In AD, activation of microglial enhancers could trigger persistent neuroinflammation, thereby exacerbating neuronal damage. The discovery and control of these cell-specific enhancers offer promising therapeutic options for precisely targeting pathological processes. Enhancer dysfunction in relation to age-related changes of gene expression is identified as an important cause of neurodegenerative diseases. The aging process alters the epigenetic landscape through both a loss of enhancer activity and an excess activation of normally inactive enhancers. These changes could affect the synthesis of genes required for neuronal maintenance and repair, hence increasing their vulnerability to degeneration. Understanding the changes in enhancer activity with age could help one to prevent diseases and provide new treatment plans [90–92].

Enhancer components confer specificity for targeting cell types or tissues and provide the precise amount required for gene regulation. In therapeutic applications—where the intent is to express genes in a particular population of cells (e.g., to deliver a therapeutic agent to neurons in neurodegenerative disorders or patients’ immune system–affected by cancer)—this is critical. Recent developments in AAV-based delivery technology complemented by enhancer-based regulatory components have produced considerable increases in both the efficacy and specificity of gene therapy [69, 93].

Significant effects on aging and neurodegenerative diseases are evident from the fact that ERTs (Enhancer Targeting RNAs) as an experimental treatment have been shown not only to alter gene expression involved in disability from both Parkinson’s Disease (PD) and Alzheimer’s Disease (AD), but also to support non-literal interpretations of the meaning of life [94]. Alzheimer’s disease, Parkinson’s disease and Huntington’s disease are all complex neurodegenerative disorders that involve the gradual loss of neuronal cells in the central nervous system over time due to multiple factors. These factors include genetics, environmental influences and epigenetic mechanisms. One of the processes associated with neurodegenerative disease progression is enhancer function, which refers to regulatory sites on DNA that modulate gene expression to maintain neuronal function. When enhancers become dysregulated, they may initiate and drive the progression of neurologic disease. In AD, enhancer dysfunction has been linked to the abnormal expression of genes relevant to neuronal survival and synaptic function, including those linked to amyloid precursor protein (APP) and tau proteins, which are key contributors to AD’s pathology. Furthermore, in neurodegeneration, enhancer components that control inflammatory responses, including microglial activation, have been identified as essential [95].

In tissue-specific and disease-context-specific gene therapy, enhancers control gene expression. New genetic engineering techniques have tested the use of Enhancer-based Gene Expression Systems in combination with viral vectors like Adeno Associated Virus for the treatment of diseases requiring Cell-Type Specificity (such as Alzheimer’s Disease) and precise targeting of cells. In addition, enhancers are being explored for cell reprogramming and differentiation into tissue/ organ types in regenerative medicine applications, particularly in the context of tissue regeneration and repair resulting from Neurodegeneration [96].

Long-term enhancer activity suppression caused by some neurodegenerative conditions (such as Alzheimer’s from Parkinson’s) can result in altered gene expression related to neuronal viability, synaptic plasticity, and neuroinflammation. In addition, excessive production of inflammatory cytokines from aberrantly activated enhancers may further worsen neurodegeneration. Therefore, in order to utilize enhancer-based gene therapy effectively, it is critical to determine the appropriate therapeutic vector and the enhancer targeted approach [97]. New viral capsids and non-viral delivery systems, such as lipid nanoparticles, could increase the effectiveness and safety of these drugs, enabling their application in conditions other than neurological ones [98].

To sum up, in neurodegenerative diseases, enhancers play an undervalued yet crucial role in regulating gene expression. Some researchers believe that dysregulation can lead to abnormal levels of gene expression, therefore supporting the cause of many diseases, such as Alzheimer’s disease (AD), Parkinson’s disease (PD), and Huntington’s disease. Current research on enhancer biology has opened new avenues for better understanding the molecular mechanisms of neurodegeneration. By identifying genes and pathways that are associated with enhancers, researchers will be able to develop more targeted and personalized treatments that address the underlying causes of these diseases. The emerging field of enhancer-based therapies has the potential to transform the way these serious diseases are diagnosed, prevented, and treated in the future.

The biological investigation of enhancers has offered insight into their possible use as therapies for use in the clinic. Enhancers can provide precise control of gene expression and that raises hope for a therapeutic approach to treat neurodegenerative diseases. In addition, researchers are now applying enhancers (i.e., researcher engineered enhancers) as part of their gene therapy programs due to advancements that have been made in the area of gene therapy and are using preclinical studies to determine if enhancer-driven approaches may be therapeutically applicable as well. Currently, there are many ongoing clinical trials that study the use of enhancer-based gene therapy to evaluate how well they can be used to regulate disease-relevant biological pathways (i.e., by using enhancers to regulate gene expression). This information provides valuable information about the feasibility, safety, and efficacy of enhancer-based therapies in the treatment of neurodegenerative diseases.

The NCT00087789 Clinical Trial (CERE-110) was designed to test the safety (tolerability) and effectiveness of the experimental gene therapy CERE-110 in patients with mild, moderate, or severe dementia due to Alzheimer’s Disease (AD). CERE-110 uses an adeno-associated virus (AAV2) to deliver a DNA sequence coding for nerve growth factor (NGF) directly into the nucleus basalis of Meynert (NBM), which is part of the human brain that is significantly impacted by AD. It was hypothesized that administration of NGF to the NBM would improve both the health and function of cholinergic neurons, which are drastically reduced in patients with AD, resulting in improvements in cognitive function. Twelve subjects received CERE-110 bilaterally through stereotactic guided injection into the NBM; based on their assigned dosage, some received 4 injections while some received 6 injections using a dose-escalation design. Each subject was observed for 24 months following the CERE-110 treatment to assess both safety and biological effects. The results demonstrated that all dosages of CERE-110 were well tolerated through laparoscopically guided injection and no significant adverse events were associated with the gene therapy. Also, PET scans of glucose metabolism indicated stabilization of glucose metabolism in the brain cells of treated subjects thus potentially indicating a decreased rate of neuronal loss. These positive findings led to the initiation of a Phase 2 clinical trial to evaluate the efficacy of CERE-110 in treating a much larger number of AD patients.

The NCT05040217 trial is a Phase I open-label safety and tolerance study of the delivery of an AAV2-BCNF gene via stereotactic/surgical delivery to the entorhinal cortex for early onset Alzheimer’s Disease (AD) and Moderate Cognitive Impairment (MCI), as well as the initial efficacy of AAV2-BCNF in subjects with either AD or MCI. The Gene Therapy Center at UC San Diego and Case Western Reserve University (Cleveland, OH) are working together on this study where they will only have 12 patients recruited and followed for 24 months. The investigators will measure the safety of the surgeries using protocols of neuroimaging, clinical testing, and laboratory studies, while the cognitive and biomarker measurements will measure efficacy. The goal of this therapy is to minimize neuronal cell death by supporting neuronal cell survival and promoting synaptic plasticity through the activity of the BDNF gene. If successful, this study will provide valuable insight into the efficacy of enhancer-based gene therapy in treating neurodegenerative disorders.

Homozygous APOE4 people with AD were the subject of a Phase I gene therapy clinical trial NCT03634007. A major inherited risk factor for AD, APOE4, the study aimed to evaluate the safety and effectiveness of a gene therapy approach meant to change APOE expression or function. Using a viral vector-based delivery system, the therapy sought to change disease progression by targeting APOE-related pathways. Though exact findings are not yet available, the completed study focused on safety assessments, cognitive outcomes, and biomarkers. This result indicates a significant improvement in gene-based treatments for AD, hence enabling the creation of focused, disease-modifying drugs for high-risk populations.

A Phase I study evaluating the safety and feasibility of NGF gene therapy for patients with mild to severe AD, the clinical trial NCT00017940 To increase the survival of cholinergic neurons, which are vital for memory and cognition, the trial called for NGF-expressing fibroblasts being implanted into the basal forebrain. The results showed that some individuals showed a slowing in cognitive deterioration and that the drug was well-tolerated with no major negative effects. Postmortem analysis revealed neuroprotective effects. Though the experiment showed no clinical effectiveness, it provided important new information on AD neurotrophic factor gene therapy and set the path for further studies in this area.

Primarily targeting APOE4 homozygotes, the clinical trial NCT05400330 focuses on long-term follow-up of participants who have received gene therapy for AD. A genetic variation, especially connected to an increased likelihood of developing AD is APOE4. The purpose of this study is to evaluate the long-term clinical outcomes and the clinical efficacy, safety and untoward effects of using gene therapy on a previously identified group of subjects (high-risk subjects). These individuals were previously treated using gene therapy directed at pathways related to Alzheimer’s Disease (AD) and this study is primarily to follow-up on the long-term clinical outcomes from the prior intervention including any additional benefit and/or harm from the prior treatment. The study will provide additional information related to the long-term clinical outcomes associated with this drug as well as provide additional data related to its ability to alter the course of disease in subjects with this specific genetic mutation.

A randomized, controlled trial called NCT00876863 evaluated the safety and effectiveness of CERE-110, a gene therapy that supplies nerve growth factor (NGF) to the brain, in the treatment of mild to moderate Alzheimer’s Disease (AD). One primary purpose of this study was to evaluate the ability of gene therapy to enhance cognitive function and to decrease the symptoms of Alzheimer’s Disease by promoting the survival and growth of neurons in the areas of the brain affected by the disease.

Evidence was collected from subjects in both the treated and untreated groups regarding the use of CERE-110 during injection to the entorhinal cortex. The outcome of this study provided data on the feasibility of gene therapy as a potential treatment for Alzheimer’s Disease and for other neurological diseases. The main purpose of the clinical trial NCT04133454 is to study the safety and tolerability of Libella gene therapy for patients with Alzheimer’s Disease. Human telomerase reverse transcriptase (hTERT) is a gene that may have a neuroprotective effect against Alzheimer’s Disease by enhancing cell viability and possibly repairing damaged neurons; it has been delivered via a viral vector (AAV). This trial includes monitoring adverse events and assessing tolerability of the medication in subjects with Alzheimer’s Disease. While the trial is currently in an uncertain state, this research is significant in exploring gene therapy methods aimed at reducing the underlying cellular damage associated with Alzheimer’s Disease.

The purpose of this research is to investigate the effects of the drug ALZ-801 on biomarkers in individuals diagnosed with early Alzheimer’s Disease (AD) who are carriers of the APOE4 gene. The clinical trial study number is NCT04693520 and intends to use amyloid-beta as a target molecule, as this protein is known to play a role in the progression of AD, particularly in individuals with the APOE4 allele which is associated with an increased risk of developing AD. Through the course of this research effort, we hope to gain insight into the potential of ALZ-801 to modify the disease’s progression in AD patients who have the APOE4 gene; assess the drug’s effect on neuronal damage biomarkers such as tau and amyloid; determine the relationship between APioE4 genotype and risk of developing AD; and help illustrate the clinical benefit of treating early-stage Alzheimer’s patients with AD. Although new volunteers are not currently being enrolled into the study, the study is ongoing, and the results from this study could provide us with additional evidence of the efficacy of ALZ-801 in early-stage AD patients who are APOE4 positive.

The gene therapy product AVB-101 is being assessed for safety and efficacy in a clinical trial of people diagnosed with an inherited form of frontotemporal dementia (FTD), also called FTD-GRN (NCT06064890). The GRN gene mutation is responsible for the build-up of a toxic neuroprotein (progranulin) in the cells of the brain. Recruitment is still open for this study, which evaluates whether AVB-101 can repair and restore normal progranulin levels, thereby treating people with FTD-GRN at the underlying level through gene therapy. Our primary goal is to evaluate the safety, tolerability, and possible clinical benefit of this gene therapy approach, which would establish a new option for treatment of this severe form of frontotemporal dementia.

Clinical trials for AD have permitted tremendous advances in gene therapy, especially in treating genetic defects and reinstalling key protein activities; parallel endeavors are now transferring to PD (PD). Like Alzheimer’s disease, PD is a neurodegenerative disorder marked by complex, illness-specific pathways and hereditary factors including the dysfunction of alpha-synuclein proteins. It is vital to look at how clinical trials in PD are using comparable molecular approaches to evaluate the possibility of enhancer-based therapies, viral vectors, and gene editing technologies to slow down or reverse the progression of PD as we move from the success stories in AD gene therapy. These studies are crucial for improving tailored therapy and improving treatment outcomes for patients with PD.

Under evaluation is the therapeutic use of gene therapy to treat patients with Parkinson’s Disease (PD). Currently, there is a randomized clinical trial involving GDNF therapy called NCT04167540 that is not accepting any participants or patients as of October 2023. This research project is designed to evaluate the effect of GDNF on the safety and efficacy as a potential treatment to either slow the course of the disease or improve motor function in Individuals diagnosed with PD. GDNF is a neurotrophic factor that may increase the survival of dopaminergic neurons, which are lost or injured in patients with Parkinson’s Disease. The aim of this trial is to evaluate the use of gene therapy for administering GDNF utilizing an adeno-associated viral vector to achieve the delivery of GDNF to the brain in a concentrated manner. Gene therapy will provide the ability to deliver the neurotrophic factor directly to dopaminergic neurons in order to prevent further cellular death as well as induce repair and regeneration of the damaged cells.

This clinical trial NCT00195143 has been completed and evaluated the safety of gene therapy directed to the subthalamic nucleus (STN) of patients diagnosed with Parkinson’s Disease (PD). In particular, this project aimed to determine whether patients could safely be administered gene therapy by using viral vectors to target the STN, thus changing the behavior of the neurons of the STN which control limb movement and may ultimately produce positive impacts on motor function and the patient’s use of levodopa (or other medications). The primary goal was to assess the adverse events and the general safety of gene therapy treatment. Although the clinical trial has been completed, additional studies need to be conducted to investigate the potential long-term effect of this type of therapy on patients with PD, as well as exploring the potential application of this type of therapy for use in other types of neurological disorders.

The clinical study NCT00643890 that was designed to assess the safety and benefits of gene therapy (AAV-GAD) using an adeno-associated viral vector (AAV) for delivery of the GABA-producing enzyme (GAD) into the subthalamic nucleus (STN) of individuals with Parkinson’s disease (PD) has since been terminated prematurely. Though the actual purpose of this technique was to increase GABA production (through AAV-GAD) to lessen excessive motor activity in the STN as part of the symptoms associated with PD, there was little information provided about the overall study and it terminated early; thus potentially indicating some issues related to either the conduct of the study regarding patient safety or efficacy, as well as potentially any other issues.

The clinical trial NCT00252850 has finished evaluating the safety of CERE-120 (AAV2-NTN) in people with idiopathic Parkinson’s disease. A gene therapy produced from an adeno-associated virus (AAV), CERE-120 is meant to send the neurturin (NTN) gene to the brain, so increasing the survival of dopaminergic neurons and so improving motor function in Parkinson’s disease (PD) sufferers. The experiment sought to assess the safety of the procedure and the effectiveness of the treatment in reducing neurodegeneration related to Parkinson’s disease. Though more study is required to confirm its effectiveness and long-term influence on illness development, CERE-120 was considered mostly safe.

The clinical trial NCT01973543 finished looking at the safety of AADC gene treatment (VY-AADC01) for people with Parkinson’s disease. VY-AADC01 uses an adeno-associated virus (AAV) vector to send the aromatic L-amino acid decarboxylase (AADC) gene to the brain, therefore increasing dopamine production in Parkinson’s disease sufferers. The experiment mostly assessed the safety of the operation and the effectiveness of the therapy in bringing dopamine levels back to normal in the brain. The results showed that the gene treatment was safe and well-tolerated, with possible benefits in improving motor function. More research is required to establish the long-term efficacy or therapeutic use of AADC gene therapy as a treatment option for patients with Parkinson’s disease. A current clinical trial enrolling patients with intermediate stages of Parkinson’s disease is NCT06285643, The REGENERATE-PD Project, which will assess the use of an adeno-associated virus (AAV) vector as a delivery system for GDNF (glial cell line-derived neurotrophic factor) directly into the brain. GDNF is a neurotropic factor that has been shown to enhance survival and function of dopaminergic neurons that are affected by Parkinson’s disease. The goal of this project is to determine whether AAV2-GDNF will increase motor function and slow the progression of the disease in patients with early-stage Parkinson’s disease. This study will also investigate how AAV2-GDNF injections in the brain will affect ongoing treatment outcomes.

### Enhancer-based gene therapy for cardiovascular diseases (CVDs)

Gene therapy treatments based on enhancers are emerging as viable options in the treatment of cardiovascular and immunologic conditions. Enhancer-mediated gene expression has a role in influencing immune responses in patients with autoimmune disease and dysregulated immune systems. Furthermore, in the case of ischemic heart disease, enhancers can potentially are being investigated to increase expression of genes that lead to tissue repair and regeneration for use in the management of cardiovascular disease [99].

Cardiovascular diseases (CVDs) are a serious global health challenge, affecting millions around the world and constituting disorders such as coronary artery disease, heart failure and stroke. Increased availability of classical therapies (pharmaceutical and surgical) for treating CVD has resulted in an increased need for additional, effective therapeutic approaches. Gene therapy provides an alternative therapeutic approach to CVD through targeting the molecular mechanisms that lead to the development of these diseases: gene therapy has emerged as a viable option for treating the underlying biological causes of the various forms of CVD. Gene therapy that employs enhancer regions, which regulate gene expression in an extremely specific and context-dependent manner, represents one of the newest and most innovative approaches in the use of gene therapy for the treatment of CVDs. Gene therapy approaches that utilize enhancers to treat CVDs may provide correction of genetic defects, as well as modified gene expression; thus, there is a potential to restore normal heart function and improve a patient’s prognosis [100, 101].

### The role of enhancers in cardiovascular disease

Enhancers are functionally defined as regulatory regions of untranslated DNA that modulate gene expression levels within a specific cell or tissue context in response to developmental or environmental cues. Enhancers are thus central to regulating gene expression in cardiovascular disease, including during normal heart development, vascular remodeling, and the physiological response to tissue stress or injury. Therefore, they can be activated by multiple types of stimuli, including growth factors, cytokines, and mechanical forces, to modulate key vascular smooth muscle cell functions, angiogenesis, and myocardial cell development [102]. Cardiovascular disorders caused by problems related to how well enhancer activity is controlled include arrhythmias, atherosclerosis, and congestive heart failure [103]. Heart failure is typically associated with alterations in the transcription of genes encoding proteins involved in myocyte function, contractility, and viability; it is the inability of the heart to pump blood effectively. Regulating these genes with enhancer elements may help restore proper gene expression patterns and, therefore, enhance the functional efficiency and regenerative capabilities of the heart [104]. The regulation of vascular disease (e.g., atherosclerosis) through the complex interactions of inflammatory mediators, smooth muscle cells, and endothelial cells has been recognized for some time. In addition to this, a therapy using enhancers which alter genes responsible for response to specific stimuli, may also be an appropriate tool to treat vascular diseases [105].

The main benefit of enhancer-based gene therapy is its ability to control gene expression with exceptional tissue specificity. In cardiovascular diseases, enhancer sequences can activate genes that promote angiogenesis, cardiac muscle regeneration, or reduce vascular inflammation. In ischemic heart disease, characterized by reduced blood flow to the myocardium, which causes tissue damage, the use of enhancers to increase pro-angiogenic genes such as VEGF (vascular endothelial growth factor) could help create new blood vessels and restore oxygen supply to the damaged tissue [106]. Additionally, enhancer treatments may stimulate the expression of genes that promote healthy healing of injured heart tissue, thereby reducing scarring and improving heart function following myocardial infarction [101].

### Strategies for enhancer-based gene therapy in cardiovascular diseases

Scientists have explored several methods for delivering enhancer-based gene therapies to heart tissue. The most effective way to deliver enhancer components into cells is via viral vectors, such as adeno-associated viruses (AAVs) and lentiviruses. Because they are non-integrating and exhibit low immunogenicity, AAVs are excellent candidates for gene therapy; thus, they may have broad applicability [107]. In contrast, various non-viral approaches (for instance, nanoparticles and electroporation) are being evaluated because they may have a more efficient method for delivering products without the risk of integrating into genes (by accident) [108].

Researchers applying CRISPR/Cas9-based epigenome-editing tools represent a markedly different approach to modifying enhancer patterns. CRISPR systems can enable precise activation or repression of specific genes via enhancers. Because no changes have occurred to the actual DNA sequence within the genome, these tools are ideal for controlling gene expression in response to disease conditions [109]. For example, it is possible to use CRISPR/Cas9 to deactivate the genes responsible for the development of cardiac hypertrophy or the fibrosis condition, as well as to reactivate the genes that allow for cardiac muscle cell survival and growth during heart failure [110].

Furthermore, synthetic biology has made it possible to design customized enhancers with distinct characteristics. Engineered synthetic enhancers can respond to specific signaling molecules, including hormones and growth factors, thereby helping to regulate gene expression under specified conditions. In the field of cardiovascular diseases, the activation of some genes in response to post-myocardial infarction or exercise-induced stress may be beneficial [111].

Although still in the early stages of clinical use, enhancer-based gene therapy shows great promise for treating heart diseases. In addition to characterizing problems with enhancer-based strategies (e.g., vector delivery optimization, design of efficient enhancer sequences, and ensuring the long-term safety and efficacy of treatment), many other problems persist despite the developments to date in this area. Recent developments have enabled clinical studies assessing the use of enhancers to medically modify gene expression (gene therapy) and to investigate how altering enhancer activity regulates important genes associated with cardiovascular disease (e.g., heart failure, atherosclerosis, or ischemic heart disease). These clinical studies will provide information relevant to future developments in the use of enhancers, particularly those aimed at individualizing therapy and enhancing treatment efficacy.

Registered as NCT01002430, the clinical trial looked into endocardial vascular endothelial growth factor D (VEGF-D) gene therapy for severe coronary heart disease (CHD). The project aimed to evaluate the safety, tolerability, and efficacy of VEGF-D gene therapy in individuals with severe coronary artery disease. Perhaps by boosting blood flow and reducing symptoms associated with coronary heart disease (CHD), the treatment was a VEGF-D gene administered to promote angiogenesis in the damaged cardiac tissue. The study examined how gene therapy affected the progression of cardiac disease and cardiac function.

Currently looking for volunteers, active clinical trial NCT06898307 Entitled “Utility of Gene Test Analysis for Diagnosis, Prognosis, and Treatment of Patients with Genetic Arrhythmic Heart Disease: The ARRHYTHMIC GENE-HEART,” this study aims to evaluate the relevance of genetic testing in the diagnosis and management of hereditary arrhythmic heart disease. The trial aims to examine how genetic sequencing may improve understanding of disease etiology, predict patient outcomes, and inform treatment plans. Through genetic testing, the study aims to provide individualized therapy for patients with arrhythmic heart disorders, thereby improving prognosis and fine-tuning treatment strategies.

A longitudinal follow-up study for Fabry Disease patients who previously received lentiviral gene therapy in the AVRO-RD-01-201 research, NCT04999059. The initiative was shelved. The study aims to evaluate the long-term safety and efficacy of lentiviral gene therapy as a potential treatment for Fabry disease. The trial would have examined the impact of gene therapy on disease progression, as well as potential adverse events and long-term consequences. The study’s findings suggest that this trial will not yield additional data.

A finished clinical study, the EXACT Trial (Epicardial Delivery of XC001 Gene Therapy for Refractory Angina Coronary Treatment), NCT04125732. The experiment aimed to evaluate the safety and efficacy of epicardial administration of XC001 gene therapy in patients with refractory angina, a condition characterized by chest discomfort resulting from inadequate blood supply to the heart despite appropriate treatment. The trial sought to determine whether XC001 may improve symptoms and increase myocardial perfusion in patients unresponsive to conventional treatments. The trial findings could clarify the feasibility of gene therapy for the management of major heart diseases, such as refractory angina.

### Enhancer-based gene therapy for cancer

**Fig. 6:**
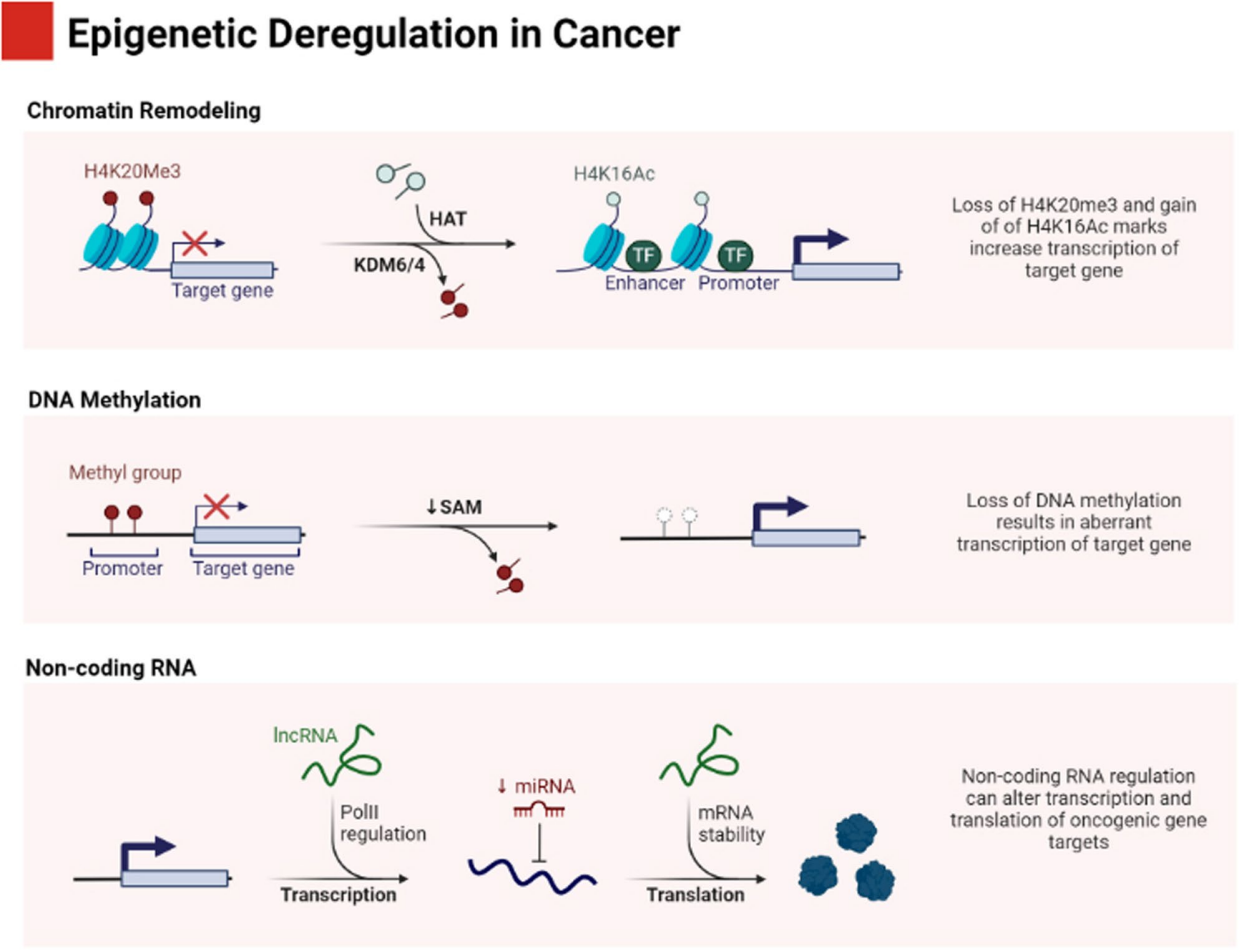
Eigenetic disregulation in cancer. The remodeling of chromatin, including the alteration of histones, and the action of histone-modifying enzymes (like histone acetyltransferases and lysine demethylases) through various enzymes (KDM4, KDM6, etc.) also affects enhancer and promoter accessibility. Examples of how to introduce activating marks (H4K16ac) or decrease repressive marks (H4K20me3) will increase the likelihood of a transcription factor binding to an enhancer or promoter and turn on transcription. The amount of successful binding depends on the environment of the cellular region where binding occurs. Changes in DNA methylation states (hypomethylation of promoters) can affect the ability to transcribe, and this ability can be altered due to imbalances of S-adenosylmethionine levels that affect DNA methylation. Therefore, long non-coding and micro-RNAs modulate transcription and post-transcriptional regulation by regulating RNA polymerase II, mRNA stability, and translation efficiency. Dysregulation of the above-mentioned epigenetic and RNA-mediated processes can result in aberrant activation of oncogenic pathways by changing the structure that allows communication between enhancers and promoters [113]

A multifarious disease shaped by several genetic, epigenetic, and environmental factors, including aberrant enhancer activity, cancer is. Although they don’t directly code for proteins, enhancers are very important in controlling gene expression, and they may be targets for cancer therapy. Mutations, chromosomal rearrangements, and epigenetic changes often affect enhancer regions, leading to oncogene overexpression and suppression of tumor suppressor genes. Abnormal activation of a remote enhancer linked to the MYC gene can cause amplification of the oncogene MYC. In some cancers, enhancer-mediated activation has been linked to other oncogenes, including BCL6, KRAS, and EGFR [112] (Fig. 6).

Enhancers in cancer biology suggest that targeting these regulatory components might provide a more particular therapeutic approach than conventional therapies like chemotherapy and radiation, which often impact fast-dividing cells and can cause significant toxicity and resistance. One approach is to develop small molecules that block the binding of transcription factors to specific enhancers, thereby inhibiting oncogene activity. Targeting MYC in cancers linked with its enhancer-driven overexpression might be a good treatment [114].

Epigenetic therapies designed to modulate enhancer function through chromatin mark modification offer a promising new avenue in cancer treatment. To reverse epigenetic changes, histone deacetylase inhibitors (HDACi) and DNA methyltransferase inhibitors (DNMTi) have been used, thereby potentially reactivating tumor suppressor genes or inhibiting oncogenes [115, 116]. In addition, CRISPR/Cas9 gene-editing methods provide a viable tool for directly modifying transcription factor binding sites or transcriptional enhancers. In certain instances, utilize CRISPR/Cas9 tools to activate specific functions within tumor suppressor genes or disrupt the function of specific enhancer elements associated with oncogenes thereby providing a more targeted approach to treat individuals diagnosed with cancer [117].

Innovatively designed methods that can be used for gene therapy to enhance therapeutic gene expression may involve incorporating synthetic enhancers into the genome providing a mechanism by which to regulate the therapeutic gene and potentially inhibit tumor growth or boost immune response against cancer [118]. In addition, various RNA types, like antisense Oligonucleotides (AONs) and Small Interfering RNAs (siRNAs), are also a way to repress gene expression that is mediated by enhancers and thus another strategy to control enhancer function when treating cancer [119].

Gene regulatory networks that control cell fate, differentiation and cell proliferation play a large role in cancer development; when enhancer functions fail or malfunction, they cause the uncontrolled progression of cancer to occur at a much faster rate than normal. Enhancer-specific therapies provide a new avenue for cancer treatment by offering more predictable and less perpetually harmful modes of action than current therapies. Enhancer biology and enhancer-based therapies will revolutionize cancer treatment by providing tailored and focused treatments to patients in the near future.

In cancer, mutations in enhancers can lead to silencing of tumor suppressor genes and/or activation of oncogenes. Epigenetic alterations that occur in enhancers may be implicated in neurological disorders, such as Alzheimer’s disease, but the epigenetic impact on enhancer regions would result in changes in the expression of neuronal genes contributing to the progression of the disease [78]. Enhancer disordered function associated with dysregulated enhancer structure/activity and autoimmune diseases like RA and SLE where abnormal capacity for response in immune system due to abnormal enhanced regulation (changes in enhancer function) [120].

Therapeutic techniques let one modulate enhancer activity, which provides fresh prospects for addressing conditions linked with enhancer malfunction. To remodel enhancers in cancer and neurological diseases, small molecules altering histone acetylation or methylation can be used. Moreover, genome-editing tools such as CRISPR/Cas9 can exactly find and fix enhancer mutations, hence restoring normal gene expression and reducing disease symptoms [121].

Enhancers are the genes that determine how cells are formed and also aid in the proper development of cells during the process of differentiation. Enhancers that do not function appropriately or that have mutations in them can lead to the development of cancers, developmental abnormalities, and nervous system disorders. Super-enhancers are enhancers with a high level of activity and contribute to the identity of specific cell types, particularly cancerous cell types. Super-enhancers cause cancer by increasing the level of oncogene production as a result of super-enhancer activity. The types of alterations to genes involved in neurodegenerative diseases (e.g., Alzheimer’s Disease) correspond to the level of super-enhancer activity associated with those types of genes [122].

Enhancer use as targets for drugs could be a new way to improve human health, since enhancer use regulates the expression of genes. As such, two classes of drugs (small molecules and RNA-based therapies) are available to increase or decrease the activity of specific enhancers. The ability of drugs to modulate tumor-promoting enhancers is likely to lead to a decrease in tumor growth; therefore, these new approaches will provide benefit in cancer treatment. Additionally, restoring or replacing the functional activity of deficient enhancers may be beneficial to the therapeutic effects in improving gene expression and the clinical manifestations of disease [31, 123]. Improvements in gene-editing tools such as CRISPR/Cas9 allow exact manipulation of enhancer areas, therefore restoring or changing enhancer function with great specificity. Genetic diseases linked to enhancer mutations, such as sickle cell disease and thalassemia, could be better managed with this level of accuracy [124].

Safety also calls for careful thought in preclinical and clinical research. Gene treatments motivated by enhancers targeting oncogenes must not set off proto-oncogenes or encourage malignancy. Enhancers in immune system regulation could set off unregulated immunological responses leading to autoimmune diseases or chronic inflammation [99].

Given the growing knowledge of enhancer-mediated gene control, the next logical step in creating targeted cancer treatments is the study of clinical trials focused on enhancer-based gene therapy. These experiments seek to exactly manage enhancer activity to either lower oncogene expression or reactivate tumor suppressor genes, therefore offering a viable replacement for conventional cancer treatments as chemotherapy. Cancer therapy enhancers’ efficacy and safety are being evaluated in clinical trials. Improvements in gene-editing technologies such as CRISPR/Cas9 allow exact targeting of enhancer areas, hence enabling more customized and safer treatments. By providing more potent, tailored medications with reduced adverse effects, the continuous development of enhancer-based gene therapies in clinical trials could change cancer treatment. Specific clinical studies looking at how enhancer-based gene therapy affects cancer will be covered in this part.

By gene expression profiling, the clinical study NCT01906632 sought to predict the therapeutic success of dendritic cell-cytokine-induced killer (DC-CIK) immunotherapy for malignant tumors. Research was conducted to determine if any of the specific tumor cells that were studied provided information on potential outcomes of a DC-CIK treatment and if the gene-expression patterns of tumor samples can serve as prognostic indicators for determining whether patients potentially have a positive treatment response to DC-CIK treatment. Researchers have determined gene-expression patterns of the tumors to possibly serve as a marker for these predicted responses which will allow for better-targeted and more efficient treatments for patients. These data at the end of the study may give some insight into which characteristics will allow for future improvements in developing more effective immunotherapy treatment of patients with these characteristics based on molecular characteristics on their tumors.

The goal of the clinical study NCT02272595 was to investigate the molecular difference between cancer tumor and normal tissue in order to create a clear picture of how different therapies would affect cancer with regards to its molecular alterations so that there was a targeted approach to therapy which is more effective. The researchers wanted to create a targeted treatment regimen based on tumor and normal tissue analysis to create a plan to specifically treat cancer cells while causing as little damage as possible to good organs through genetic/molecular methods in patients with a diagnosis of cancer. The trial has now completed, and the results will create a better way to treat cancer on an individual basis through a targeted approach based on individual tumor characteristics.

NCT00403221 was a Phase I trial of RTVP-1 gene therapy for prostate cancer patients prior to radical prostatectomy. This trial evaluated initial results and safety of RTVP-1 gene therapy targeting prostate cancer cells. Gene therapy was administered prior to a radical prostatectomy procedure to measure the impact of gene therapy on the behavior of tumors and potentially improvement in outcomes of treatment. Significant findings were made at the conclusion of this study regarding the viability and safety of using gene therapy approaches for prostate cancer.

Immunotherapies that use cells, like CAR-T cells and DC-CIK approaches, are based upon the ex vivo genetic modification and cell expansion rather than on enhancer-dependent transcription regulation within cells in vivo. Endogenous gene regulation in immune cells may be influenced by enhancer elements, but the current types of CAR-T and DC-CIK therapies currently available for clinical use have not been developed with the enhancer targeting as a form of immunotherapy in mind. The technologies represented by these therapies are therefore presented in this article as advancements adjacent to the field of gene (or cellular) therapy in general and not strictly as examples of enhancer-based therapies.

Treating metastatic breast cancer with PD-1 knockdown anti-MUC1 CAR-T cells was the clinical trial NCT05812326. By removing the PD-1 checkpoint receptor, which suppresses T cell function in cancer, this project sought to improve the effectiveness of chimeric antigen receptor T (CAR-T) cell therapy. To improve the immune response against the tumor, the altered CAR-T cells aim MUC1, a protein usually found in breast cancer cells. The experiment ended by assessing the effectiveness of this complex immunotherapy technique in treating breast cancer, possibly targeting immune evasion mechanisms linked to the condition.

The clinical trial NCT00186862 investigates the use of genetically altered allogeneic neuroblastoma cells for the therapy of relapsed or refractory neuroblastoma. The trial aimed to evaluate the safety, feasibility, and potential therapeutic effects of using genetically altered neuroblastoma cells to provoke an immune response against the tumor. By changing neuroblastoma cells to increase their ability to trigger the immune system, the study sought to create a new treatment for neuroblastoma patients resistant to existing medicines. The experiment ended with notable findings on the effectiveness of gene-modified cell therapies in cancer treatment. Table 2: Summary of finished clinical trials relating to enhancer-based gene therapy for cancer, immunological diseases, cardiovascular diseases, and neurodegenerative diseases.

**Table 2:** Summarizing completed clinical trials related to enhancer-based gene therapies for neurodegenerative diseases, cardiovascular diseases, immunological diseases, and cancer

Therapy Name	Disease Target	Approach	Status	Phase
Cancer				
Gendicine	Head and neck squamous cell carcinoma	Recombinant adenovirus delivering p53 gene to induce tumor cell apoptosis	Approved	-
Talimogene laherparepvec (T-VEC)	Melanoma	Oncolytic virus engineered to express GM-CSF, stimulating immune response	Approved	-
Enadenotucirev	Various solid tumors	Oncolytic adenovirus administered intravenously to infect and lyse tumor cells	Completed	-
Ad.p53	Advanced cancers (e.g., HNSCC, NSCLC)	Adenovirus-based p53 gene therapy to restore apoptosis in tumor cells	Completed	Phase I/II
Virotherapy (VSV-G)	Melanoma, Squamous Cell Carcinoma	Modified oncolytic viruses targeting cancer cells, enhancing immune response	Completed	Phase II
BB-401	Pancreatic Cancer	Adenovirus-mediated IL-12 gene therapy, activating immune response in tumors	Completed	Phase II
Neurological Diseases				
Strimvelis	ADA-SCID (Severe Combined Immunodeficiency)	Ex vivo gene therapy using autologous CD34+ cells transduced with ADA cDNA	Approved	-
Convection Enhanced Delivery (CED)	Parkinson's Disease	Direct delivery of therapeutic agents (e.g., GDNF) into the brain to support neuronal survival	Completed	Phase I/II
Brain-Targeted AAV Gene Therapy	Alzheimer’s Disease	AAV-delivered tau protein modulating gene therapy aimed at preventing neurodegeneration	Completed	Phase I
Luxturna (voretigene neparvovec)	Inherited retinal dystrophies	AAV-based gene therapy to deliver RPE65 gene for retinal degeneration treatment	Approved	-
VY-AADC01	Parkinson's Disease	Adeno-associated virus (AAV) gene therapy for the delivery of AADC gene to improve dopamine production	Completed	Phase II
Cardiovascular Diseases				
NTLA-2001 (Nexiguran Ziclumeran)	ATTR Amyloidosis with Cardiomyopathy	CRISPR-based gene editing to reduce transthyretin production	Phase 3	-
Lenti-D	Leukocyte Adhesion Deficiency Type I	Ex vivo gene therapy using lentiviral vectors to correct LFA-1 deficiency	Completed	Phase I/II
VEGF-A gene therapy (AdVEGF)	Critical limb ischemia (CLI)	Adenoviral vector delivery of VEGF-A to promote angiogenesis in ischemic tissue	Completed	Phase II
AGT103T	Peripheral Artery Disease	Adeno-associated virus (AAV) carrying VEGF to promote blood vessel growth in patients with PAD	Completed	Phase I/II
Cardiogen-1	Myocardial infarction	Adenoviral vector-based gene therapy to deliver VEGF gene for heart regeneration	Completed	Phase II
Immunological Diseases				
CAR T-cell Therapy	Lupus	Genetically modified T-cells to target and eliminate autoreactive cells	Ongoing	Phase I/II
Zynteglo (LentiGlobin)	Beta-Thalassemia, Sickle Cell Disease	Gene therapy using lentiviral vectors to deliver functional hemoglobin genes	Approved	-
Kymriah (tisagenlecleucel)	B-cell Acute Lymphoblastic Leukemia (ALL)	CAR T-cell therapy targeting CD19+ B-cells to treat leukemia	Approved	-
Yescarta (axicabtagene ciloleucel)	Diffuse Large B-Cell Lymphoma (DLBCL)	CAR T-cell therapy targeting CD19 to treat relapsed/refractory DLBCL	Approved	-
GSK3150004	Rheumatoid Arthritis, Lupus	Investigational CAR T-cell therapies for autoimmune diseases	Ongoing	Phase II

The table shows clinical studies highlighting the many diseases and approaches handled by enhancer-based gene therapies. These treatments include cancer, neurological diseases, heart problems, and immunological concerns. While AAV vectors have shown promise in treating genetic disorders and neurological diseases, oncolytic viruses and gene editing techniques, including CRISPR and CAR T-cell therapies—have become essential weapons in the fight against cancers and immunological diseases. Regarding regulatory status, drugs like Kymriah and Yescarta have been authorized; others, including NTLA-2001 [125], are still in advanced stages of clinical testing. The continuous development of these drugs will depend on continual invention and improvement of delivery methods as well as actions to reduce safety issues. These trials highlight the growing potential of enhancer-based and gene therapy technologies in providing individualized, precision medicine for a broad spectrum of disorders, hence stressing its transformative role in future therapies

## Enhancer-based gene therapy and safety issues

Although there are high hopes for enhancer-based gene therapy, a few obstacles must be overcome – most notably the safe and reliable delivery of therapeutic enhancers. One of the huge concerns is the risk of ectopic enhancer activation and the potential for carcinogenic and off-target effects being caused. To help avoid such issues, accurate vector and delivery method selection are required. AAV, or adeno-associated virus-based vectors, are commonly used vectors for gene therapy due to their ability to target specific tissues and cell types. AAV vectors do have their efficiency limitations, particularly regarding the ability of these vectors to cross the blood-brain barrier (BBB), which is of importance in the treatment of neurodegenerative diseases, and the risk of inducing immune responses against these vectors. There are ongoing investigations into alternative delivery systems, including lentiviral vectors and lipid nanoparticles, as possible means to deliver enhancer elements. However, it continues to be a challenge to improve delivery vector designs to reduce off-target effects and increase transduction efficiency.

It is critical that the safety issues are addressed concerning the potential of unintentional activation of enhancers in unexpected genomic locations. Targeted modification of enhancer sequences using CRISPR/Cas9-mediated enhancer editing will help reduce the chances of unintended activation or repression of off-target genes. The development of sophisticated screening procedures to assess enhancer activity during therapy will also help to guarantee that only intended genes will be affected.

In summary, enhancer-based gene therapies hold a great deal of therapeutic potential, provided that the best possible delivery systems are utilized in conjunction with the minimization of safety risks. Future advancement in targeted delivery methodologies, gene editing technologies, and vector engineering will play a major role in the safety and efficacy of enhancer-based therapies.

### Challenges and possible routes in enhancer-driven gene therapy

Enhancer-based gene therapy may be a path for precision medicine, particularly in the case of neurological diseases, cancer and other complex conditions. While there is potential to accomplish this goal, the effort faces many challenges including the need to understand the biology of enhancers and to solve the many technical and legal obstructions to applying this technique clinically. This section will address these challenges and examine new opportunities to enhance the relevance and effectiveness of enhancer-based therapies.

### Variability in enhancer function in context

The context-dependent actions of enhancers is a significant hurdle in enhancer-mediated gene therapy. While enhancers are crucial in controlling the expression of genes (i.e., by redirecting the action of promoters), their effective regulation can be influenced greatly by the type and manner in which they are used in different cells, their relative tissue specificity, and by the underlying disease state. For example, an enhancer that promotes a high level of gene expression in one type of cell may produce little if any gene expression in a different type; and moreover, an enhancer’s action could be altered when the target cells are subjected to a pathogenic condition. Due to this variability, it is much more challenging to identify common enhancer components that will be relevant to multiple tissues or multiple contexts. Recent data indicate that enhancers have different activities for normal and diseased tissues, which will ultimately have a direct impact on the efficacy of a treatment [126, 127]. This challenge emphasizes the need of thorough studies on enhancer activity in certain disease settings to create focused therapies.

### Manufacturing and scalability issues

The widespread use of enhancer-based drugs is of great concern. To make gene therapies commercially available, it will be necessary to produce large amounts of viral vectors (e.g., adeno-associated viruses, or AAVs) that can carry enhancer sequences as part of the gene therapy construct. Enhancing AAV manufacturing while ensuring that the AAVs produced are of high quality, effective and safe for patients is going to be very challenging. There are many factors that may complicate the manufacture of AAV vectors containing appropriate enhancers for specific cell types or tissues. Recent studies have highlighted some of these challenges and the need for improved manufacturing techniques and rigorous purification standards [128, 129]. The widespread use of enhancer-based gene therapies will depend on addressing these manufacturing constraints.

### Regulatory frameworks and clinical translation

Enhancer based gene therapy must undergo significant regulatory evaluation in order to transition from laboratory experimentation to real world use. The FDA is working to facilitate gene therapy by creating regulatory transparency and processes around long-term safety and effectiveness of genetic therapies, including development of vector distribution mechanisms and mitigation of unintended effects. In addition to creating guidance on the safety of enhancers, including synthetic enhancers, it is incumbent on researchers to resolve the regulatory issues associated with using enhancers as a viable drug delivery mechanism [130]. Though cooperation among academics, businesses, and regulatory bodies will be vital to overcome these challenges, the biotech industry has shown increasing interest in enhancer-based gene therapies.

### Creative tools to drive the field forward

The innovative capabilities offered by creative technology such as synthetic enhancers, epigenome editing etc. are important for overcoming limitations that presently exist. Technologies that allow for epigenomic editing (e.g. CRISPR/Cas9 systems) allow for precise modification of enhancer regions, and therefore precise modulation of gene expression. This may open new avenues for therapies to specifically modulate gene expression to treat a disease condition, and could produce results that are more flexible and more accurate [131]. Also, the creation of synthetic enhancers which were designed to give stable and flexible results may lead to innovative medicine for individualized treatment of patients. Disorders that generate complex genetics, such as cancer and neurological disorders, may be improved with the use of synthetic enhancers [132].

### Single-cell multi-omics’ role in enhancer identification

Recent advancements in single cell multi-omic technologies have provided new insights into enhancer function at a higher resolution than before. As a consequence of integrating single-cell RNA sequencing, ATAC-seq and epigenetic characterization, researchers can now identify active enhancer regions within specific cell types or pathology. These tools provide detailed maps of enhancers that may serve as a guide for the development of new drugs directed at particular patient populations. Through discovering cell-specific enhancers that can be targeted in gene therapy, these technologies increase the precision and efficacy of treatment [126]. As these methods develop, they might change enhancer-based gene therapy by providing better knowledge of the control of enhancer activity in different pathological contexts.

### Customized enhancer maps for tailored patient therapies

An area of future interest with respect to enhancer-mediated gene therapy has the potential of creating personalized treatment regimens. Individualized enhancer maps indicating how enhancers operate for individual patients will ultimately provide appropriately targeted medications based on the patient’s genetic and epigenetic characteristics. Using these kinds of precision-based techniques could ultimately lead to more favorable clinical outcomes for patients by accounting for changes in enhancer function unique to each specific patient. This approach will likely prove significant in more complex disorders such as cancer, where there are often large variations in enhancer activity among the population [133]. Personalized enhancer maps could help to target particular cell types or tissues, hence improving the specificity of gene therapy and lowering off-target effects.

Enhancer-driven gene therapies hold enormous hope for the evolution of personalized medicine through Enhancer-driven gene therapy and gene regulation technologies, regardless of the difficulties faced in establishing context-dependent enhancer activity, scalability/manufacturing/regulatory issues and additional learning of enhancer biology will be a result of collaboration and cooperative efforts of all parties involved, including scientists, physicians, and industry executives. Utilizing available epigenome editing tools, creating synthetic enhancers, and applying single-cell multi-omics tools presents a multitude of exciting opportunities for investigating and advancing the utility and applications of enhancer-based therapies. Increased knowledge of enhancer biology and utilizing specific approaches toward developing enhancer-targeted therapies will allow us to develop therapeutics that are both more potent and more targeted toward specific disease states.

### Future directions in enhancer-based gene therapy

Gene therapy through enhancer-based approaches may revolutionize how we treat many different diseases. The development of synthetic enhancer technology, epigenome editing techniques, and single-cell multi-omics technologies are creating opportunities for more precise and effective methods of delivering gene therapies. Synthetic enhancers can be designed to function in a consistent and adaptable manner so that they can be used to create personalized therapeutic approaches in patients suffering from complex diseases such as cancer and other neurological disorders [134]. Moreover, single-cell multi-omics methods (comprising RNA sequencing, ATAC-seq and epigenome analysis) enable the identification of cell-type specific enhancers, which may aid in the development of targeted therapeutics [135].

Researchers can develop drugs tailored to a patient’s unique genetic and epigenetic characteristics by developing personalized enhancer maps. This improves the treatments’ specificity and effectiveness.

## Conclusion

Enhancers are key controlling, tissue-specific elements at the interface of molecular biology/genetics/epigenetics that modulate the functions of cells through gene expression regulation. Rapid advances in functional genomics stemming from high-throughput technologies and computational techniques have provided additional insight into enhancer biology (from health to disease). The use of enhancer biology provides an excellent means to study the context of gene regulation, and new technologies allow us to manipulate enhancer activity and study their potential for use in gene therapy. However, the context-dependent nature of enhancers, interindividual variability, and the difficulty of determining and measuring expression over time will limit the effectiveness of enhancer-based strategies when translating them for clinical use. Therefore, as functional genomic and epigenetic editing technologies develop, we will be able to determine whether the overall advantages of enhancer-based strategies outweigh those of established gene therapy methods. An objective evaluation of the advantages and disadvantages of enhancer-based strategies will be essential to ensure that this technology is implemented in clinical practice responsibly. Emphasizing these enhancers provides a potential treatment approach. Apart from cancer, enhancer dysregulation is linked to many genetic and neurological disorders. In neurodevelopmental illnesses include congenital heart disease, mutations in tissue-specific enhancers disrupt normal development. Altered enhancer activity in neurodegenerative diseases like Alzheimer’s can affect genes required for neuronal function and survival. The combination of artificial intelligence (AI) and multi-omics data is improving our understanding of enhancer function, hence offering new perspectives on enhancer-promoter interactions across several cell types and pathological conditions. Methods of machine learning are being used to predict interactions and activity of enhancers, hence improving treatment outcomes. Especially in complex tissues like the brain, single-cell multi-omics technologies will increase our knowledge of enhancer control. Promising tailored therapies aimed at dysregulated enhancer activity in both genetic and acquired diseases result from the ongoing evolution of enhancer-based gene therapies supported by epigenome editing and synthetic biology. The ability to precisely find and target enhancers in certain tissues and diseases will be crucial as the field develops. Emphasizing a turning point in the field of precision medicine, enhancer-based gene therapies represent a major development in tailored, effective, and safer therapeutics.

## Data Availability

All data supporting this review article are available within the paper and its Supplementary Information.
